# A protein–protein interaction map reveals that the *Coxiella burnetii* effector CirB inhibits host proteasome activity

**DOI:** 10.1371/journal.ppat.1010660

**Published:** 2022-07-11

**Authors:** Mengjiao Fu, Yuchen Liu, Guannan Wang, Peng Wang, Jianing Zhang, Chen Chen, Mingliang Zhao, Shan Zhang, Jun Jiao, Xuan Ouyang, Yonghui Yu, Bohai Wen, Chengzhi He, Jian Wang, Dongsheng Zhou, Xiaolu Xiong

**Affiliations:** 1 State Key Laboratory of Pathogen and Biosecurity, Beijing Institute of Microbiology and Epidemiology, Academy of Military Medicine Sciences, Fengtai, Beijing,China; 2 State Key Laboratory of Proteomics, Beijing Proteome Research Center, National Center for Protein Sciences, Beijing Institute of Lifeomics, Beijing, China; 3 Beijing Advanced Innovation Center for Soft Matter Science and Engineering, Beijing University of Chemical Technology, Beijing, China; 4 Department of Molecular and Cell Biology, University of California, Berkeley, Berkeley, California, United States of America; Purdue University, UNITED STATES

## Abstract

*Coxiella burnetii* is the etiological agent of the zoonotic disease Q fever, which is featured by its ability to replicate in acid vacuoles resembling the lysosomal network. One key virulence determinant of *C*. *burnetii* is the Dot/Icm system that transfers more than 150 effector proteins into host cells. These effectors function to construct the lysosome-like compartment permissive for bacterial replication, but the functions of most of these effectors remain elusive. In this study, we used an affinity tag purification mass spectrometry (AP-MS) approach to generate a *C*. *burnetii*-human protein-protein interaction (PPI) map involving 53 *C*. *burnetii* effectors and 3480 host proteins. This PPI map revealed that the *C*. *burnetii* effector CBU0425 (designated CirB) interacts with most subunits of the 20S core proteasome. We found that ectopically expressed CirB inhibits hydrolytic activity of the proteasome. In addition, overexpression of CirB in *C*. *burnetii* caused dramatic inhibition of proteasome activity in host cells, while knocking down CirB expression alleviated such inhibitory effects. Moreover, we showed that a region of CirB that spans residues 91–120 binds to the proteasome subunit PSMB5 (beta 5). Finally, PSMB5 knockdown promotes *C*. *burnetii* virulence, highlighting the importance of proteasome activity modulation during the course of *C*. *burnetii* infection.

## Author summary

As the causative agent of Q fever, *C*. *burnetii* colonizes host cells by transferring effector proteins into the host cytoplasm through its Dot/Icm secretion system to construct a replicative vacuole. The function of effectors remains largely unknown. Here, we performed a large-scale AP-MS screen to analyze the interactions among *C*. *burnetii* effectors and human proteins. These analyses found that CirB functions as an inhibitor of host proteasome activity, revealing that proteasome activity is important for intracellular survival of *C*. *burnetii*. Our data have laid the foundation for future exploring the molecular mechanisms underlying the roles of *C*. *burnetii* effectors in its virulence and for the identification of novel potential drug targets for the development of novel therapeutic treatment for *C*. *burnetii* infection.

## Introduction

*Coxiella burnetii* is a Gram-negative bacterium responsible for the worldwide zoonotic disease Q fever, which manifests with acute or chronic symptoms. The Dot/Icm type IV secretion system (T4SS) is considered a key virulence determinant of *C*. *burnetii*, which transfers effector proteins into host cells to promote bacterial survival and replication [1,2]. To date, more than 150 *C*. *burnetii* effectors have been identified using *Legionella pneumophila* as a surrogate [2–6]. Notably, the *C*. *burnetii* T4SS effector AnkG inhibits host cell apoptosis by interacting with the host proteins p32, importin-α1 and RNA helicase 21 [7,8]. In addition to AnkG, the effectors CaeA and CaeB prevent mitochondrial-dependent (intrinsic) apoptosis [9]. Three effectors, CetCb2, CetCb4, and Cem9, modulate the mitogen-activated protein kinase (MAPK) pathway in yeast [10]. Three effectors, including *Coxiella* vacuolar proteins (Cvp) A, CvpB, and CvpF, locate on the membrane of *Coxiella*-containing vacuoles (CCVs) and promote intracellular replication by interacting with host targets through distinct mechanisms [11–14]. Additionally, the effectors Cig57 and CirA subvert membrane trafficking through interactions with FCHO2 and Rho GTPases, respectively [15,16]. In recent studies, NopA and CinF have been reported to inhibit the innate immune signaling pathway by perturbing the nuclear import of transcription factors and dephosphorylating IκB, respectively [17,18]. The diverse host pathways involved in *C*. *burnetii* infection have revealed that, in addition to the most widely studied autophagy-lysosome and apoptosis-related pathways, many previously understudied pathways in host cells may play important roles in *C*. *burnetii* pathogenesis.

Since the functions of most identified *C*. *burnetii* effector proteins have not been elucidated, identifying the interaction between *C*. *burnetii* effectors and host proteins will provide important leads for our understanding of *C*. *burnetii* pathogenesis and for combating infections. Previously, yeast two-hybrid (Y2H) assays had been performed to screen host proteins interacting with *C*. *burnetii* effectors [19]. However, the PPIs identified in this screen only covered approximately 20% of the reported *C*. *burnetii* effectors, and the interactions between the other effectors and host proteins remain to be identified. Among the various high-throughput experimental methods available, mass spectrometry (MS) is a powerful approach with high coverage and accuracy for identifying new pathogen-host PPIs and for elucidating the mechanisms of pathogen replication, uncovering new functions of pathogen proteins, and revealing host pathways involved in infection [20–22]. Several host pathways required for a number of important pathogens including HIV [23], Zika virus [24], severe acute respiratory syndrome-coronavirus-2 (SARS-CoV-2) [25], *Chlamydia trachomatis* [26], and *Mycobacterium tuberculosis* [27], have been systematically identified using affinity purification-mass spectrometry (AP-MS). Similarly, studies aiming to explore the mechanisms by which *C*. *burnetii* effectors manipulate host cells using this unbiased method are necessary and meaningful.

The proteasome is one of the most important components for protein degradation in host cells. The activity of this abundant protein complex is tightly regulated and essential for the turnover of host proteins in the cytoplasm and nucleus [28] and has been shown to degrade thousands of short-lived and regulatory proteins, as well as damaged and misfolded proteins, to regulate various cellular functions [29–32]. The 20S core particle of the proteasome, which is composed of four stacked heptameric rings formed by α_7_β_7_β_7_α_7_ subunits, is a barrel-shaped cylinder in which protein degradation occurs [33]. The proteolytically active subunits β1 (PSMB6), β2 (PSMB7), and β5 (PSMB5) harbor caspase-like, trypsin-like, and chymotrypsin-like catalytic activities, respectively [34]. In pathogen-invaded host cells, proteasomes cleave viral or bacterial antigens to generate peptides for MHC class I presentation, facilitating the clearance of infected cells [35]. Accordingly, pathogens have evolved numerous strategies to hijack proteasomes and ensure their intracellular replication. For example, *L*. *pneumophila* exploits host proteasomes to target unnecessary bacterial proteins for degradation by expressing ubiquitin ligases [36,37]. However, the involvement of the proteasome in *C*. *burnetii* infection has not yet been reported.

In this study, using a combination of AP-MS and bioinformatics analysis, we identified PPIs between *C*. *burnetii* T4SS effectors and host proteins. Furthermore, we revealed that CBU0425 (designated *Coxiella* effector for intracellular replication B, CirB [6]) interacted with PSMB5 to inhibit the hydrolytic activity of the proteasome. This *C*. *burnetii*-human PPI map has advanced our understanding of the complex interactions between *C*. *burnetii* and its host, and the role of proteasomes in *C*. *burnetii* infection was first revealed.

## Results

### Generation of a *C*. *burnetii*-human PPI network

**Fig 1:**
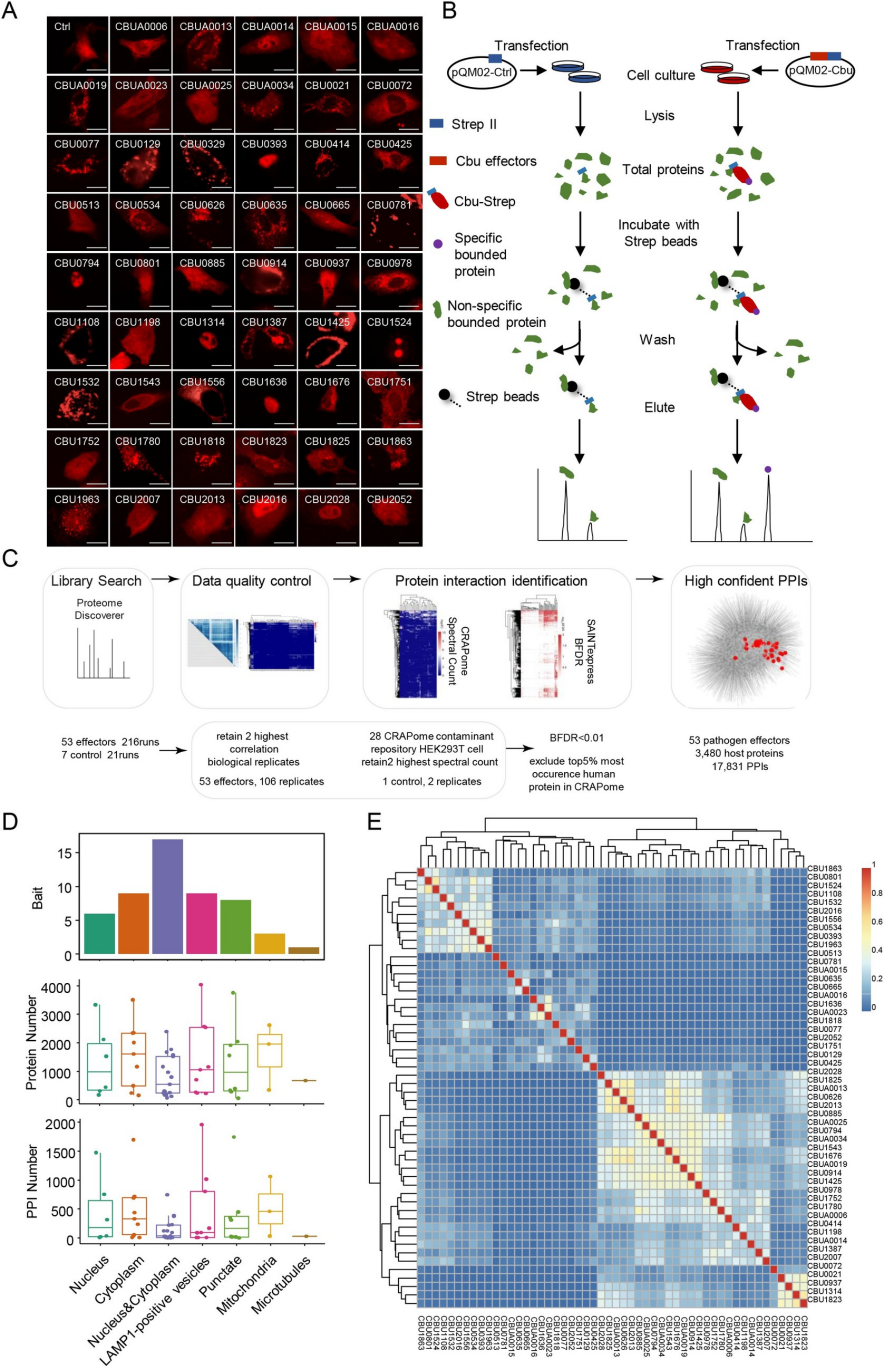
AP-MS analysis of *C*. *burnetii* effectors. (A) PQM02-cbu plasmids were transfected into HeLa cells, and the expression of mCherry-Strep-tagged effectors was observed under a fluorescence microscope (magnification, ×600, bar = 20 μm). (B-C) Schematic diagram of affinity tag purification (B) and MS data analysis (C). (D) Number of host proteins and PPIs that identified by the AP-MS approach based on the subcellular location of effectors. (E) The Jaccard index of *C*. *burnetii* effectors.

To date, more than 150 effector proteins of *C*. *burnetii* have been identified, and some of these effectors have been reported to manipulate diverse host signaling pathways to promote *C*. *burnetii* replication, but the functions of the majority of these effectors remain elusive [1,2,38]. To further explore the biological functions of these effectors, an AP-MS approach was used to identify potential physical interactions between *C*. *burnetii* effectors and host proteins. Eighty genes encoding effectors from the *C*. *burnetii* Nine Mile phase II (NMII) strain identified from 2010 to 2018 [3–6,12,39–42] were selected and individually cloned into a pQM02 vector with an N-terminal mCherry and C-terminal twin-Strep tag (pQM02-cbu), and 53 of them were expressed at high levels in human cervical cancer (HeLa) cells (Fig 1A). These ectopically expressed effectors showed different subcellular localizations, with some colocalizing with LAMP1-positive vesicles (S1A Fig). To perform the AP-MS, effector proteins were enriched in human embryonic kidney (HEK-293T) cells with Strep-Tactin Sepharose beads and identified by liquid chromatography with tandem mass spectrometry (LC-MS/MS) (Fig 1B). The results were then processed using the method shown in Fig 1C. Raw data were searched using Proteome Discoverer, and the Pearson’s correlation coefficients (PCC) between three biological replicates of each effector was calculated separately. Only data from two replicates with the highest PCC were used for further analysis. Furthermore, only the data from the replicates with a PCC greater than 0.6 were used for interaction identification. Effectors with a low correlation or with a low identified protein number were rid of for MS samples. Thus, the final 106 raw data of 53 effectors were selected from 216 runs, including controls. The number of proteins identified in the mCherry-Strep controls ranged from 17 to 640, with a median of 83. To obtain a high confidence PPI network, a high-quality negative control is needed. Two highest spectral counts of all 28 HEK-293T cell experiments in the CRAPome database were used as controls. Therefore, 53 effectors and 2 CRAPome controls were first scored with the SAINTexpress algorithm. Host interactions with a Bayesian false discovery rate (BFDR) less than 0.01 (in 19,761 potential interactions) were subjected to further filtering. Then, the proteins that appeared most frequently in all human samples (443 proteins occurring in the top 5th percentile, S1 Table) were arbitrarily excluded. All these proteins appeared in at least 87 experiments in all 343 datasets from CRAPome. The *C*. *burnetii*-human PPI network contained 17,831 PPIs consisting of 53 T4SS effectors and 3,480 host proteins. The statistical figure of the total protein number identified by MS and the PPI number involving each effector protein were shown in Fig 1D. The effectors were classified according to the subcellular localization we identified in this study or reported previously [5,7,43]. The PCC of biological replicates of each effector ranged from 0.66 to 0.97 (S1B Fig). The number of interactions ranged from 3 to 1,955. Then, we selected 26 PPIs of interest derived from AP-MS experiments for further study and verified 13 of them in a co-immunoprecipitation (co-IP) assay (S2 Fig).

The Jaccard coefficients of *C*. *burnetii* effectors were calculated to evaluate the similarity between host proteins (Fig 1E), and the results showed that effectors could be classified into 5 clusters according to the similarity of their binding partners. However, we found that the clusters of these effectors did not exactly match the subcellular localization previously reported. This difference may have been a result of the protein expression profiles of different cell lines or a considerable number of cross-subcellular localization of protein interactions. Another potential explanation for this difference is the nonbiologically relevant interactions between proteins from different cellular compartments in lysed cells [44]. Nevertheless, the PPI network would provide a comprehensive reference for the in-depth study of uncharacterized effectors.

### Systematic analysis of the protein interaction map

**Fig 2:**
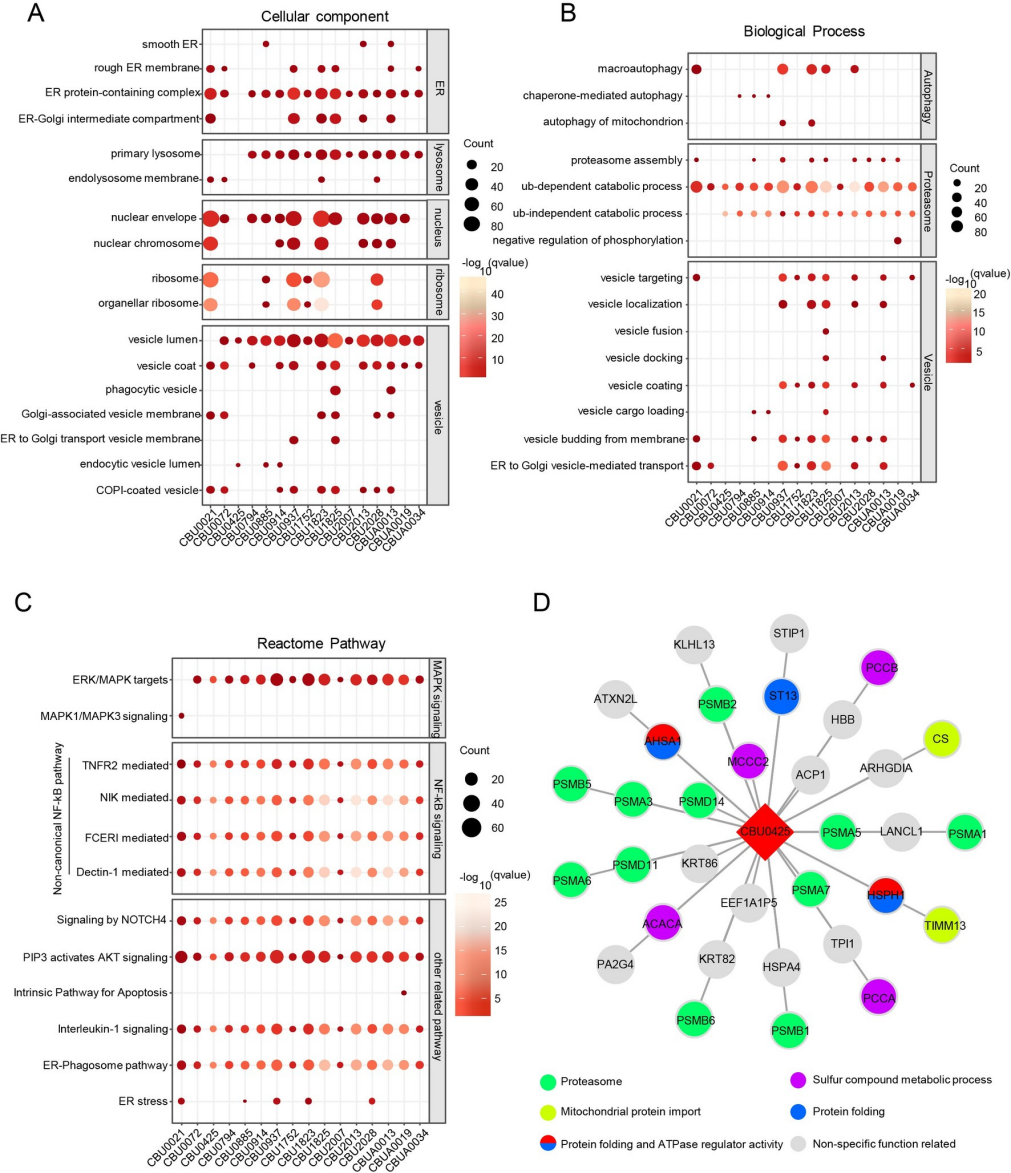
Systematic analyses of the *C*. *burnetii*-host PPI network. (A-C) Functional characterization of clustered PPIs. (A) Cellular components, (B) Biological processes, (C) Reactome pathways. (D) Interactions between CirB and host proteins.

To further define the function of these *C*. *burnetii* effectors, Gene Ontology and pathway enrichment analyses (Reactome) of each effector were performed separately. Cell component annotations of host interactors were noted first. As shown in Fig 2A, the endoplasmic reticulum, lysosome, nucleus, ribosome and vesicle were the main organelles in which these effectors were localized. As expected, the significantly enriched terms of host interactors were autophagy, proteasome and vesicle-related (Fig 2B). The effector proteins (CBU0021, CBU0937, CBU1823, CBU1825 and CBU2013) were mainly associated with autophagy. Protein catabolism mediated by the ubiquitin-dependent proteasome was another enriched term that was hit by more than 15 *C*. *burnetii* effectors. Additionally, some of these *C*. *burnetii* effectors exhibited a clear preference for the process involved in vesicle trafficking. Signaling pathways, mainly the MAPK, NF-κB and AKT pathways, were also enriched (Fig 2C). Surprisingly, the noncanonical, but not the canonical, NF-κB pathway was noticeably enriched, suggesting a potential role for certain effectors in modulating host inflammatory response through this signaling pathway. In addition, the main interactions enriched in functional pathways, such as vesicles (S3A Fig), NF-κB pathway (S3B Fig) and apoptosis (S3C Fig), were also analyzed. Among the enriched effectors, CirB (CBU0425) was mainly enriched in the process of proteasome-mediated degradation, differing from effectors that play multiple roles, as indicated by their enrichment in the cellular component and biological process categories. Furthermore, the interaction map revealed that CirB interacts with the majority subunits of the 20S core proteasome (Fig 2D), suggesting its role in modulating proteasome function.

### Ectopically expressed CirB inhibits the activity of the host proteasome

**Fig 3:**
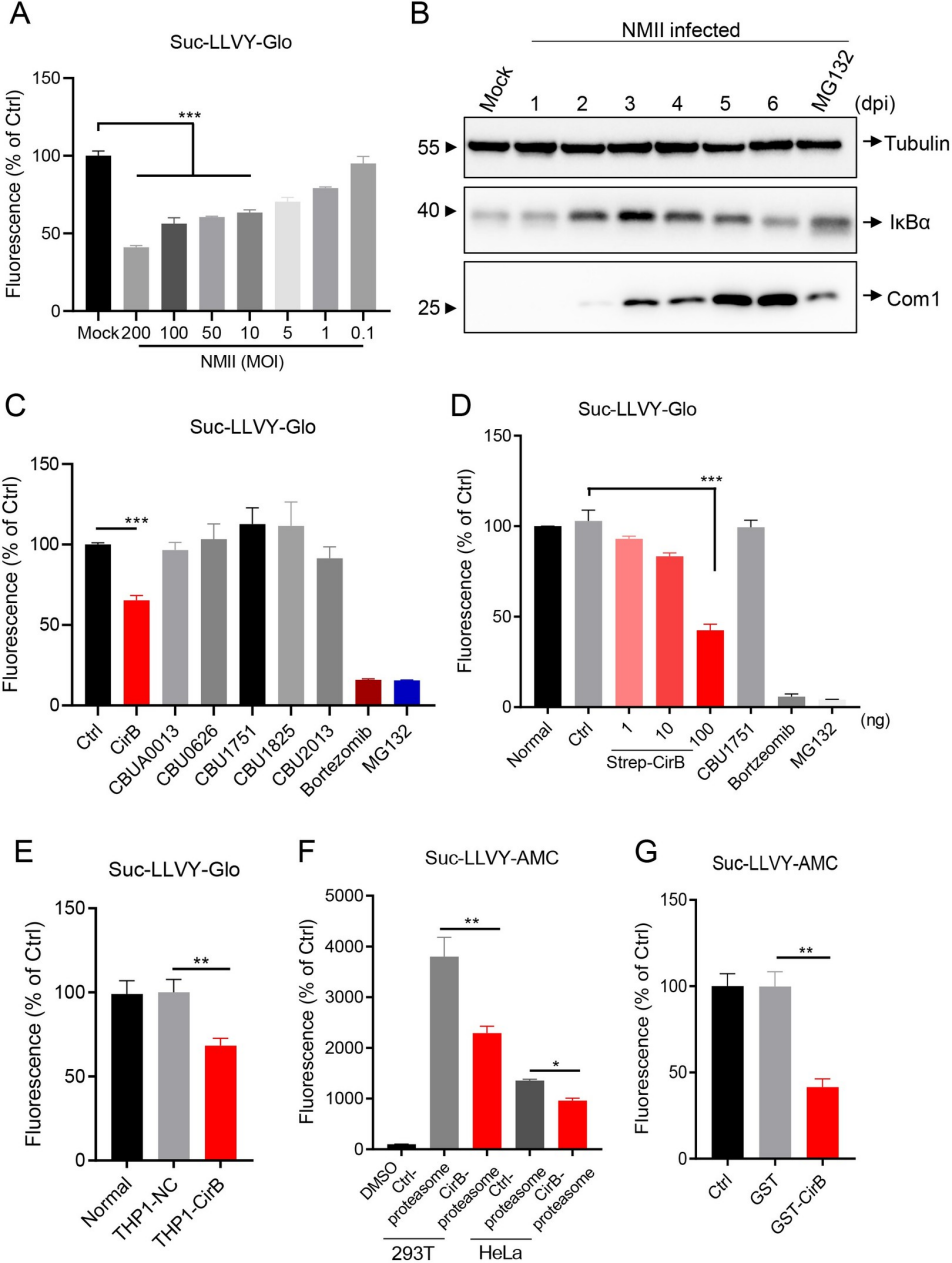
CirB inhibits proteasome activity. (A) THP-1 cells were differentiated with PMA (200 nM) in a 96-well plate and infected with NMII strains at different MOIs or left uninfected. At two days post-infection, proteasome activity was measured using a proteasome-Glo cell-based assay. (B) Differentiated THP-1 cells were infected with the NMII strain at an MOI of 100. At the indicated time points post-infection, cells were lysed and the expression of IκBα, Tubulin and Com1 was detected using Western blotting with the indicated antibodies. A sample collected from cells treated with MG132 (10 μM, 8 hours) on day 2 post-infection served as a control. (C) Plasmids expressing the indicated effector proteins were transfected into HeLa cells. Twenty-four hours later, the proteasome activity of the cells was measured as described above. The cells treated with bortezomib (10 μM, 8 hours) and MG132 (10 μM, 8 hours) served as positive controls. (D) Different amount of CirB, 100 ng of CBU1751, or 100 ng of the control plasmid was transfected into HeLa cells, respectively. Then, the proteasome activity was measured 24 hours later. (E) Normal THP-1, THP1-NC or THP1-CirB cells were differentiated with 200 nM PMA. Proteasome activity of the cells were measured 48 hours later. (F) PQM02-CirB or control plasmids were transfected into 293T or HeLa cells, proteasomes were enriched from cells, and the hydrolytic activity of proteasomes degrading the Suc-LLVY-AMC substrate was detected in vitro. (G) The 1mM of GST-CirB or GST proteins expressed in prokaryotes was added to the system containing 20S proteasome, 0.1% SDS and Suc-LLVY-AMC substrate in Tris-HCl buffer. The fluorescence was measured at an excitation wavelength of 390 nm and an emission wavelength of 460 nm. Data are representative of three independent experiments and bars represent the mean ± SD. *, *p* < 0.05, **, *p* < 0.01, and ***, *p* < 0.001.

Although *C*. *burnetii* has been reported to manipulate diverse host processes and signaling pathways, no information showing the relationship between *C*. *burnetii* and the host proteasome has been reported. In our study, we first found that the hydrolytic activity of the proteasome decreased during *C*. *burnetii* infection, while cell viability was not significantly affected (Figs 3A and S4A), and the expression level of IκBα, which was mainly degraded by the proteasome, was significantly higher than that in the uninfected group (Fig 3B). As revealed by the PPI network, the proteasome subunits were targeted by several *C*. *burnetii* effectors; hence, we wondered whether any of these effectors was involved in inhibiting proteasome activity during infection. As shown in Fig 3C, only ectopically expressed CirB significantly inhibited proteasome activity, and this inhibitory effect presented a dose-dependent manner in the case of CBU1751 as a control (Figs 3D and S4B and S4C). In THP1-CirB (CirB overexpressing) cells, proteasome activity was also significantly lower than that in control cells (Fig 3E). To investigate proteasome activity in vitro, proteasomes of cells overexpressing CirB or a control plasmid were purified and subjected to a functional assay using Suc-LLVY-AMC as a substrate. Whereas proteasomes isolated from control cells possessed high hydrolytic activity (S4D Fig), proteasomes similarly purified from cells overexpressing CirB exhibited significantly lower activity (Fig 3F). Moreover, GST-CirB purified from E. *coli* also inhibited substrate degradation by the 20S proteasome (Fig 3G). Collectively, these results indicated that CirB inhibits the activity of the host proteasome.

### CirB is involved in inhibiting proteasome activity during *C*. *burnetii* infection

**Fig 4:**
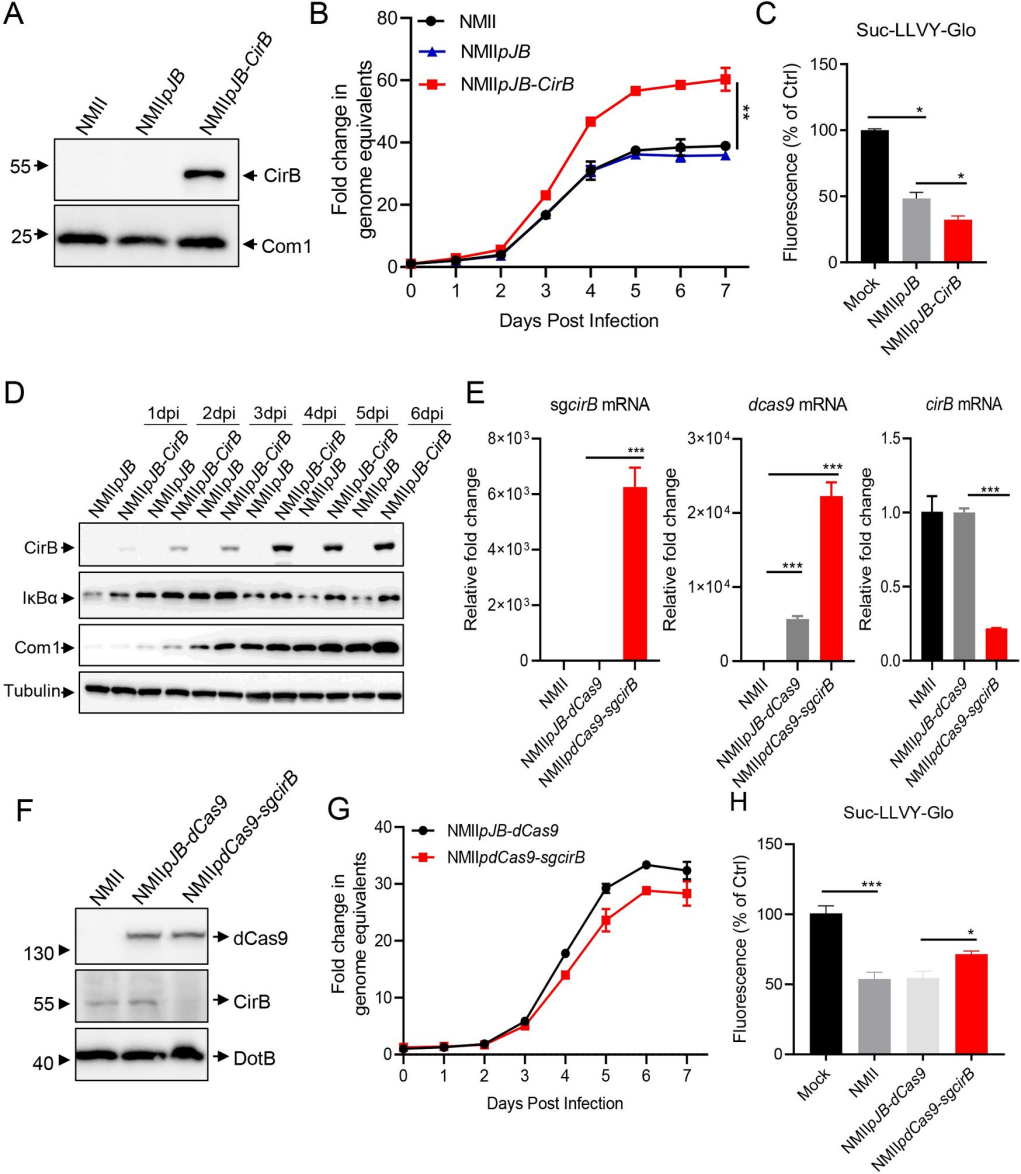
Overexpression or knockdown of CirB in NMII affects proteasome activity. (A) Differentiated THP-1 cells were infected with NMII, NMII*pJB* or NMII*pJB-CirB* at an MOI of 100. Three days later, cells were lysed, and the expression of CirB or Com1 was examined using Western blotting with the indicated antibodies. (B) Differentiated THP-1 cells were infected with NMII*pJB* or NMII*pJB-CirB* at an MOI of 20. At different time points post-infection, the total DNA was extracted, and the GE of *C*. *burnetii* was quantitated using qPCR. Fold change in GE was calculated by comparing the GE at different days post-infection to day 0. Data are representative of three independent experiments and bars represent the mean ± SD, **, *p* < 0.01. (C) Differentiated THP-1 cells were infected with the indicated *C*. *burnetii* strains at an MOI of 100 for 2 days. Then, proteasome activity of the infected cells was measured. (D) Differentiated THP-1 cells were infected with NMII*pJB* or NMII*pJB-CirB* at an MOI of 100. At different times post-infection, the expression of IκBα and CirB were examined using the indicated antibodies. The expression of Tubulin and Com1 was used as the internal control for host cells and *C*. *burnetii* respectively. (E) Differentiated THP-1 cells were infected with NMII, NMII*pdCas9* or NMII*pdCas9-sgcirB* at an MOI of 100. Two days post-infection, total RNA was extracted and the mRNA levels of sg*cirB*, *dcas9* and *cirB* were quantitated using qRT-PCR. The relative gene expression was calculated as follows: the mRNA levels of sg*cirB*, *dcas9* and *cirB* in NMII*pdCas9*-or NMII*pdCas9-sgcirB*-infected cells/that in NMII-infected cells. The expression of *rpoB* was used as an internal control. (F) Differentiated THP-1 cells were infected with NMII, NMII*pJB-dCas9* or NMII*pdCas9-sgcirB* at an MOI of 100. Two days later, cells were lysed and the expression of CirB, dCas9 and DotB were examined using Western blotting with the indicated antibodies. (G) Differentiated THP-1 cells were infected with NMII*pdCas9* or NMII*pdCas9-sgcirB* at an MOI of 20. At different time points post-infection, the total DNA was extracted and the GE of *C*. *burnetii* was quantitated using qPCR. (H) Differentiated THP-1 cells were infected with different strains at an MOI of 100. Two days later, the proteasome activity of infected cells was measured. Data are representative of three independent experiments and bars represent the mean ± SD. *, *p* < 0.05, **, *p* < 0.01, and ***, *p* < 0.001.

CirB has been shown to be a T4SS effector of *C*. *burnetii* based on the fact that it can be translocated into host cells by *L*. *pneumophila* or *C*. *burnetii* in a Dot/Icm-dependent manner [5,6]. In this study, we validated the translocation of CirB by *C*. *burnetii* by performing a β-lactamase-based translocation assay (S5A and S5B Fig). To further explore the role of CirB in modulating proteasome activity during *C*. *burnetii* infection, we first attempted to generate a *cirB* deletion mutant through homologous recombination [45] but were unable to obtain such mutants despite extensive efforts. Therefore, we sought to employ alternative strategies to alter the expression of CirB in *C*. *burnetii* and detect the subsequent effects of this altered expression on host proteasome activity. The *cirB* gene was cloned into a pJB-Kan-3×FLAG plasmid and introduced into the NMII strain via electroporation to generate a strain that stably overexpressing CirB (NMII*pJB-CirB*, Fig 4A). The growth characteristic of NMII*pJB-CirB* in acidified citrate cysteine medium-2 (ACCM-2) was not different from that of the control strain NMII*pJB* (S5C Fig), while NMII*pJB-CirB* showed better intracellular replication in infected THP-1 cells (Fig 4B). Regarding proteasome activity, NMII*pJB-CirB* infection caused a detectable inhibition of proteasome activity compared to strain NMII*pJB* (Fig 4C). Meanwhile, the degradation of IκBα in cells infected with NMII*pJB-CirB* was also inhibited due to the overexpression of CirB (Fig 4D). Using a modified CRISPRi system (S5D Fig), we successfully constructed strain NMII*pdCas9-sgcirB*, and the mRNA levels of *dcas9*, *cirB* and single guide RNA (sgRNA) targeting *cirB* were verified using qRT-PCR. As shown in Fig 4E, the expression of *dcas9* and sgRNA was highly induced and the mRNA level of *cirB* was decreased by approximately 90% compared to that of the control strains NMII and NMII*pdCas9*. Moreover, CirB expression in THP-1 cells infected with NMII*pdCas9-sgcirB* was almost completely suppressed, as determined by Western blotting (Fig 4F). The growth of NMII*pdCas9-sgcirB* in THP-1 cells or ACCM-2 was not significantly different from that of the control strains (Figs 4G and S5E), but NMII*pdCas9-sgcirB* infection partially alleviated the inhibition of proteasome activity induced by *C*. *burnetii* infection (Fig 4H). The primers used for detection are listed in S5F Fig.

### CirB interacts with PSMB5

**Fig 5:**
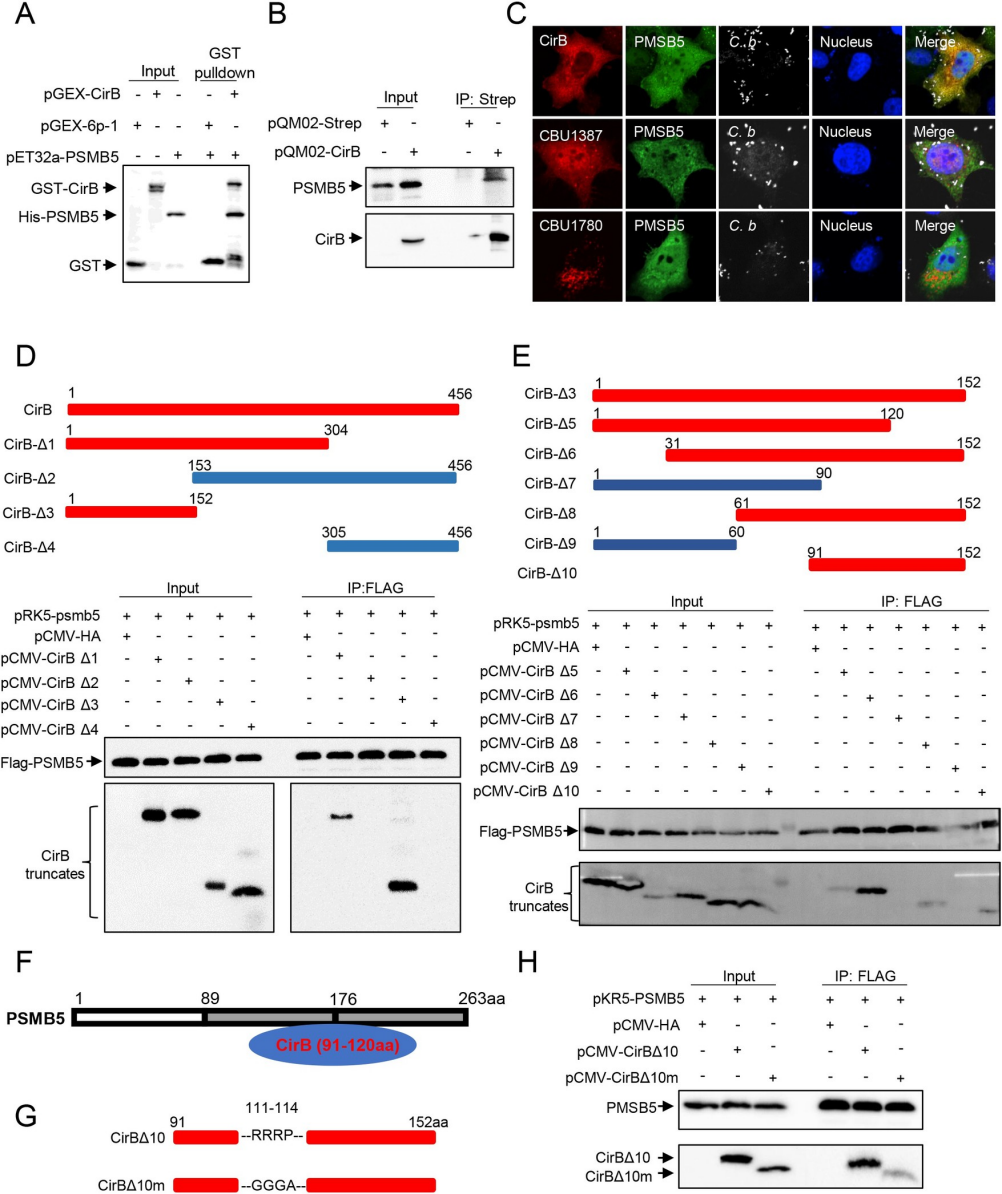
CirB interacts with the host protein PSMB5. (A) pGEX-CirB, pGEX-GST or pET32a-PSMB5 was separately expressed in *E*. *coli* BL21. The purified GST-CirB or GST was co-incubated with GST Sepharose beads, then HIS-PSMB5 was subjected to the GST-conjugated beads. After eluting, samples were loaded for Western blotting. (B) HEK-293T cells were transfected with pQM02-CirB or the corresponding control plasmid. Then, the cells were lysed and proteins were immunoprecipitated with an anti-Strep antibody, and the intracellular PSMB5 was immunoblotted with an anti-PSMB5 antibody. (C) HeLa cells were transfected with mCherry-tagged CirB, CBU1780 or CBU1387 before *C*. *burnetii* infection at an MOI of 100, respectively. Two days later, cells were fixed, and endogenous PSMB5 was probed with an anti-PSMB5 and a corresponding goat anti-rabbit Alexa Fluor 488 antibody (green). *C*. *burnetii* was stained with anti-*C*. *burnetii* serum, followed by a Cy5 conjugated goat anti-mouse IgG H&L antibody (white). The nucleus was stained with DAPI (blue). Images were captured using a confocal microscope (magnification, ×600, bar = 20 μm). (D-E) FLAG-PSMB5 and HA-tagged CirB truncation mutants were co-transfected into 293T cells, and the cell lysates were immunoprecipitated with an anti-FLAG antibody and immunoblotted with an anti-HA or anti-FLAG antibody. Schematic diagram of the truncation mutants of CirB is shown in the upper panel. Sections marked in red represent the truncation mutants responsible for the interaction. (F) Schematic diagram showing the regions of PSMB5 and CirB interaction. (G) The diagram of the mutation sites in CirBΔ10m. (H) PRK5-PSMB5 and pCMV-CirBΔ10, pCMV-CirBΔ10m or pCMV-HA were co-transfected into 293T cells, and the cell lysates were immunoprecipitated with anti-FLAG antibody and immunoblotted with anti-FLAG or anti-HA antibody.

CirB was identified to interact with several functional subunits of the human 20S proteasome, including PSMB5, which displays a chymotrypsin-like activity. We therefore focused on the interaction of CirB and PSMB5 and sought to identify the key domain critical for this interaction. HIS-PSMB5 could be pulled down with GST-CirB expressed in prokaryotes in vitro (Fig 5A), and overexpressed CirB also interacted with endogenous PSMB5 in HeLa cells (Fig 5B), which confirmed the interaction between these proteins. Furthermore, in NMII-infected cells, overexpressed CirB colocalized with endogenous PSMB5 with another two effector proteins served as controls (Fig 5C). Because CirB lacks an obvious known PPI domain or motif, we constructed truncation mutants of CirB and PSMB5 to determine the key regions responsible for their interaction. CirB interacted with all PSMB5 truncation mutants, except for PSMB5Δ3, while the binding between CirB and PSMB5Δ4 or Δ5 was weaker, indicating that PSMB5 interacts with CirB through both amino acid regions 89–176 and 177–263 (S6A and S6B Fig). Simultaneously, amino acids 1–152 of CirB interacted with PSMB5, which was verified by co-IP and laser confocal microscopy (Figs 5D and S6C). We further eliminated portions of the CirB amino acid 1–152 sequence to generate 6 truncation mutants and ultimately identified CirB amino acids 91–120 as the key region mediating the interaction of CirB with PSMB5 (Figs 5E, 5F and S6D). As proline-and arginine-rich peptides have been reported to be flexible allosteric modulators of the proteasome and to exert the inhibitory effects [46,47], we replaced the RRRP sequence (amino acid residues 111–114 of CirB) in the CirBΔ10 truncation mutant to GGGA and thus generated CirBΔ10m. As shown in Fig 5G and 5H, the interaction of PSMB5 and CirB Δ10 was not abolished but was reduced when the RRRP sequence was substituted.

### A region that spans residues 91 to 120 of CirB is sufficient to inhibit proteasome activity

**Fig 6:**
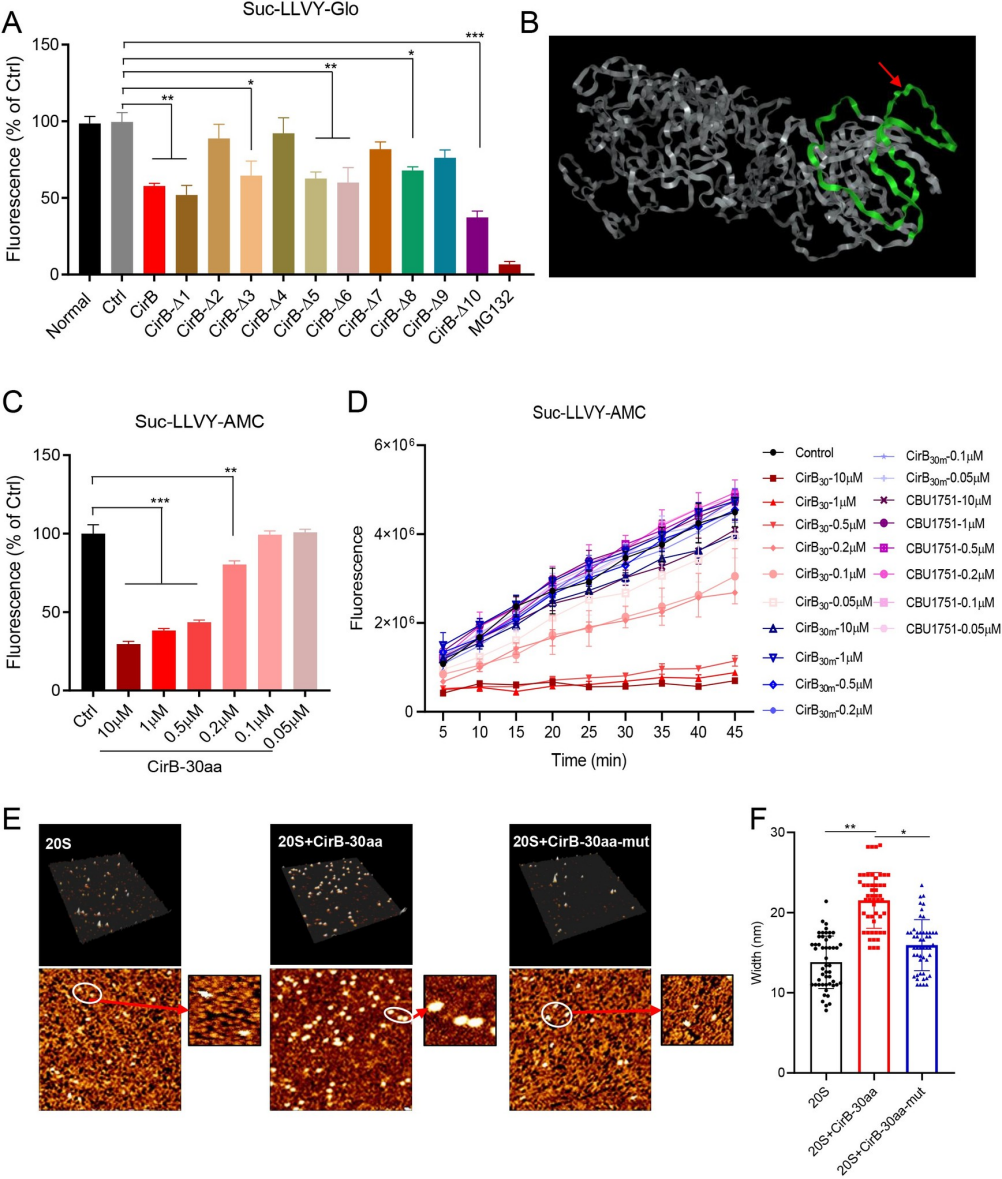
The 91–120 aa region is the key domain of CirB that inhibits proteasome activity. (A) Plasmids expressing CirB or CirB truncation mutants, as well as the control plasmid, were transfected into HeLa cells. Twenty-four hours later, the proteasome activity of cells was measured. Data are representative of three independent experiments and bars represent the mean ± SD. **, *p* < 0.01, and ***, *p* < 0.001. (B) The predicted 3D protein structure of CirB protein. The sequence marked in green was the 91–120 amino acid region of CirB, and the amino acids “RRRP” were indicated with a red arrow. (C) Different concentrations of synthetic CirB-30aa were added to the mixture containing 20S proteasome, 0.1% SDS and Suc-LLVY-AMC substrate in Tris-HCl buffer. The fluorescence was measured at an excitation wavelength of 390 nm and an emission wavelength of 460 nm. (D) The effects of CirB-30aa, CirB-30aa-mut and CBU1751-30aa peptides on proteasome activity were examined, and the fluorescence was measured every 5 min. (E) The effects of CirB-30aa on proteasomes were observed with AFM. The purified and unmodified human 20S proteasomes and 20S proteasomes treated with CirB-30aa or CirB-30aa-mut were electrostatically attached to the mica substrate, and areas ranging from 0.09 (right panel) to 1 μm^2^ (left panel) were scanned. Upper panel: representative 3D images of a 1 μm× 1 μm field of 20S proteasomes in height mode. Lower panel: representative images of 20S proteasomes scanned using AFM. (F) The average size of the proteosome was altered by CirB-30aa. The width of purified and unmodified human 20S proteasomes and 20S proteasomes treated with CirB-30aa or CirB-30aa-mut was measured and calculated. n = 50. Data are representative of two independent experiments and bars represent the mean ± SD of 50 proteasomes. *, *p* < 0.05, and **, *p* < 0.01.

To determine the CirB region that mainly exerted the inhibitory effect on proteasome activity, CirB and its truncation mutants were individually transfected into HeLa cells, and proteasome activity was assessed at 24 hours post-transfection. CirBΔ5 and CirBΔ10 exerted a significant inhibitory effect on proteasome activity (Fig 6A), and their overlapping sequence was amino acids 91–120 of CirB, which was also the region required for PSMB5 binding, as explained above. The predicted structure of CirB obtained by AlphaFold2 [48] was visualized using Web3dmodel (https://web3dmol.net/). As shown in Fig 6B, the 91–120 amino acid region of CirB, which is critical for the interaction of CirB and PSMB5, is marked in green. In the predicted structure, this region appears to fold independent of other regions of the protein, suggesting that residues 91–120 of CirB are important for CirB function. We thus synthesized a peptide of CirB_91-120aa_ (designated CirB-30aa) and measured its impact on proteasome activity. In Tris-HCl buffer, CirB-30aa significantly inhibited 20S proteasome-mediated substrate degradation in a dose-dependent manner (Fig 6C). We next hypothesized that the proline and arginine residues in CirB-30aa might be the key residues driving the inhibitory effects of CirB-30aa. A CirB-30aa mutant peptide (in which RRRP was replaced with GGGA, designated CirB-30aa-mut) and a 30 aa peptide of CBU1751_88-113aa_ (designated CBU1751-30aa) were synthesized and used as controls to confirm the inhibitory effect of CirB-30aa (S6E Fig). The results of the verification experiment showed that CirB-30aa exerted an inhibitory effect on proteasome activity in a dose- and time-dependent manner, while CirB-30aa-mut and CBU1751-30aa failed to affect proteasome activity (Fig 6D). To determine whether CirB-30aa functioned as an allosteric inhibitor of 20S proteasome activity, atomic force microscopy (AFM) imaging was used to detect conformational changes of the 20S proteasome with or without exposure to CirB-30aa. Purified 20S proteasome exhibited a cylindrical shape with an average width of 13.81 nm in the field of 300×300nm, and the addition of CirB-30aa clearly caused an increase in the 20S proteasome size, with the width of the proteasome increasing to 21.52 nm (Fig 6E and 6F). However, CirB-30aa-mut exerted no obvious effect on the size of the 20S proteasome. These results suggested that the 91–120 aa of CirB is the functional region critical for CirB-mediated inhibition of proteasome activity.

### Interference of proteasome activity or PSMB5 expression promotes intracellular replication of *C*. *burnetii*

**Fig 7:**
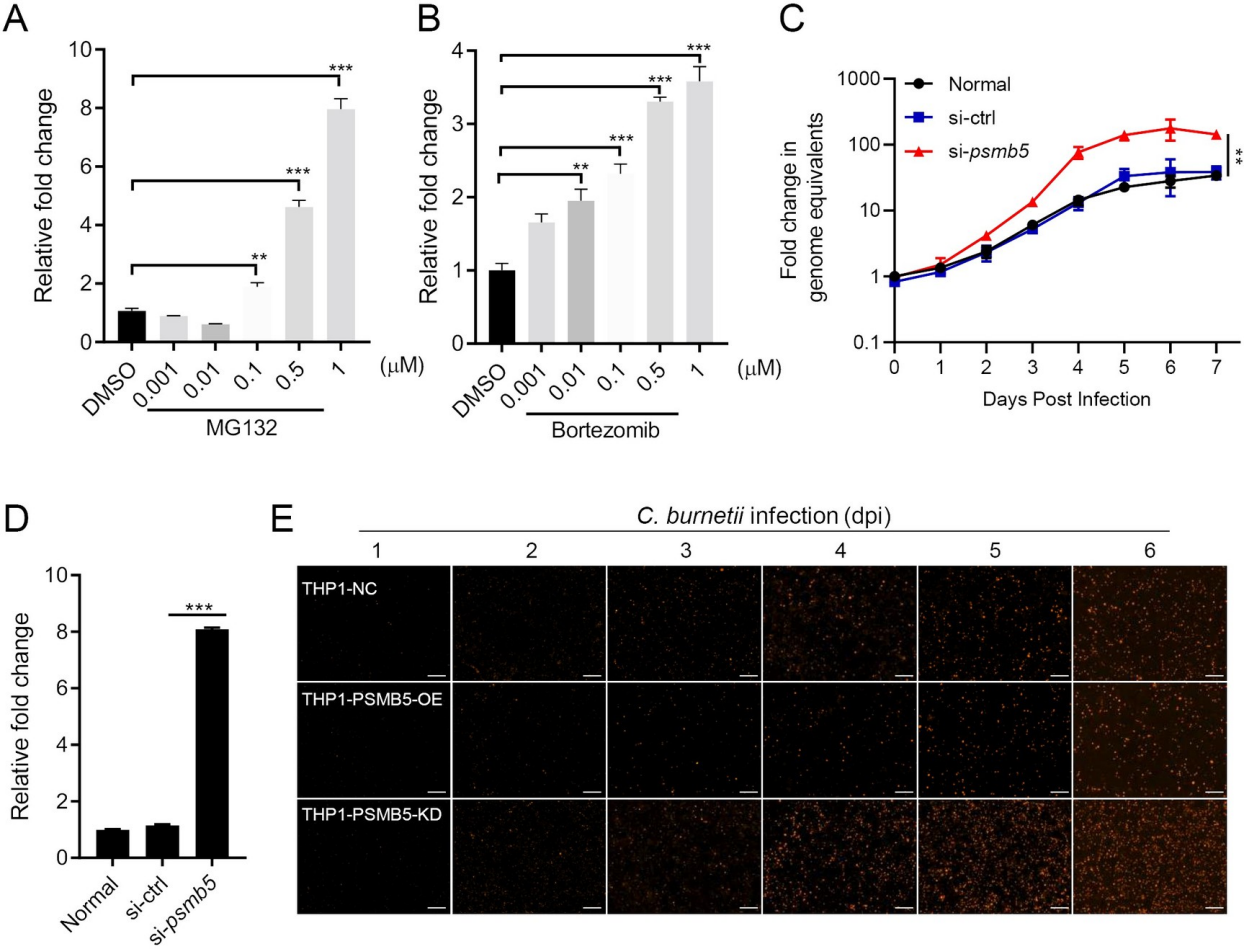
Inhibition of proteasome activity or PSMB5 expression promotes the intracellular replication of *C*. *burnetii*. (A-B) Differentiated THP-1 cells were infected with *C*. *burnetii* at an MOI of 100. Four hours post-infection, cells were washed and cultured with 10% RPMI1640 containing different concentrations of MG132 (A) or bortezomib (B) for 6 hours. Then, the cells were washed and cultured for another 2 days before being collected for DNA extraction. The GE of *C*. *burnetii* was quantitated using qPCR. The relative fold change was calculated as follows: the GE of *C*. *burnetii* in MG132-or bortezomib-treated cells/that in DMSO-treated cells. (C) Si-*psmb5* or control siRNA was transfected into HeLa cells. Four hours post transfection, the cells were infected with *C*. *burnetii* at an MOI of 20. The GE of *C*. *burnetii* was quantitated using qPCR at the indicated time points after infection. Fold change in GE was calculated as follows: GE at different days post-infection compared to day 0. Data are representative of three independent experiments and bars represent the mean ± SD, **, *p* < 0.01. (D) Differentiated THP-1 cells were transfected with si-*psmb5* or control siRNA. Then, the cells were infected, and the GE of *C*. *burnetii* was quantitated at four days post-infection. The relative fold change was calculated as follows: GE of *C*. *burnetii* in si-ctrl-or si-*psmb5*-transfected cells/that in normal cells. (E) THP1-NC, THP1-PSMB5-OE (cells overexpressing PSMB5) or THP1-PSMB5-KD (PSMB5 knockdown) cells were differentiated and infected with NMII*pKM230* (mCherry) at an MOI of 20. The replication of NMII*pKM230* in cells was observed under a fluorescence microscope (magnification, ×100, bar = 100 μm). Data are representative of three independent experiments.

As one of two major pathways for protein degradation in host cells, the proteasome plays an important role in maintaining cell homeostasis. To explore the relationship between proteasome function and *C*. *burnetii* replication, we treated *C*. *burnetii* infected THP-1 cells with MG132, a widely used proteasome inhibitor, and bortezomib, a selective 20S proteasome inhibitor that targets the catalytic sites of PSMB5, at different concentrations for 6 hours. Two days after treatment, total DNA was extracted, and the genome equivalent (GE) of *C*. *burnetii* DNA was quantitated using qPCR. The inhibition of proteasome activity by MG132 or bortezomib promoted the intracellular replication of *C*. *burnetii* in a dose-dependent manner (Fig 7A and 7B). Moreover, PSMB5 knockdown in cells using a short interfering RNA (siRNA) increased the replication of *C*. *burnetii* (Figs 7C, 7D and S7A). To visualize this effect more clearly, THP-1 cells were infected with a mutant strain-NMII*pKM230* [3], which was capable of stably expressing the mCherry fluorescence protein. More intense mCherry fluorescence was observed in the THP-1 cell line with PSMB5 stably knockdown (THP1-PSMB5-KD), while the mCherry fluorescence intensity was weaker in THP-1 cells stably overexpressing PSMB5 (THP1-PSMB5-OE), indicating the negative effect of PSMB5 on the intracellular replication of *C*. *burnetii* (Figs 7E and S7B–S7E).

**Fig 8:**
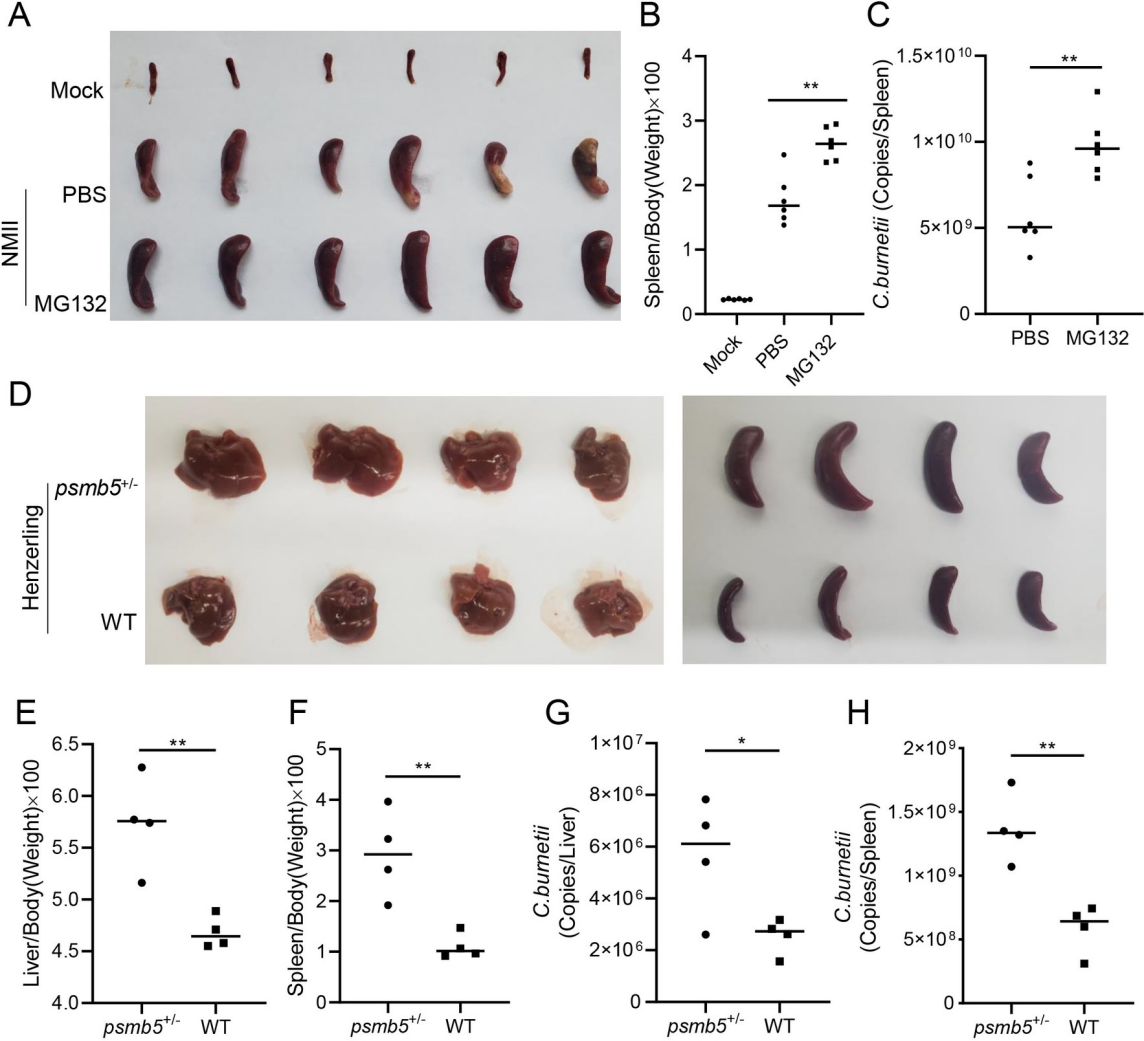
Inhibition of proteasome activity promotes the replication of *C*. *burnetii* in mouse models. (A) Mock-or *C*. *burnetii*-infected SCID mice were sacrificed at day 14 post-infection. The spleens of mice were removed and photographed after bloodletting. (B) The ratio of spleen weight to body weight of each mouse was calculated. (C) The GE of *C*. *burnetii* in spleens was determined by qPCR. The data are presented as the mean of n = 6 mice per group, and the standard error is indicated by the error bar. (D) C57BL/6 J wild-type mice or *psmb5*^*+/-*^ mice were sacrificed at 14 days post-infection. The spleens and livers were removed and photographed. (E-F) The ratio of liver weight (E) or spleen weight (F) to body weight of each mouse was calculated. (G-H) The GE of *C*. *burnetii* in livers (G) or spleens (H) was quantitated using qPCR. The data are presented as the mean of n = 4 mice per group, and the standard error is indicated by the error bar.

Because avirulent *C*. *burnetii* NMII strains are unable to establish successful infection in immunocompetent mice [49], an SCID mouse model was further employed to explore the relationship between proteasome function and the replication of NMII strains. SCID mice were pretreated with or without MG132 for two weeks before being infected with NMII strains, and the degree of splenomegaly and bacterial load were evaluated at 14 days post-infection (S8A and S8B Fig). Compared with PBS, MG132 treatment alone had no effect on the generation of splenomegaly in the uninfected mice (S8C Fig). In mice infected with NMII strains, severe splenomegaly was observed, and the degree of splenomegaly of the mice treated with MG132 was greater (Fig 8A and 8B). In addition, the bacterial load in the spleens of the mice treated with MG132 was significantly higher than that of the untreated mice, as determined using a qPCR assay (Fig 8C). Because virulent *C*. *burnetii* phase I strains have full-length LPS and can successfully infect and propagate in immunocompetent mice [50], C57BL/6J-wild-type (WT) and C57BL/6J-*psmb5*^*+/-*^ mice (S8D Fig) were used to study the relationship between PSMB5 function and the replication of *C*. *burnetii* virulent strains. In C57BL/6J-*psmb5*^*+/-*^ mice experimentally infected with Henzerling phase I strain, more severe splenomegaly and hepatomegaly were observed (Fig 8D), and the spleen-to-weight ratio and liver-to-weight ratio of *psmb5*^*+/-*^ mice were significantly higher than those of the WT mice (Fig 8E and 8F). Furthermore, the bacterial load in the spleens and livers of *psmb5*^*+/-*^ mice was significantly higher than that of WT mice (Fig 8G and 8H). These results indicated that the inhibition of proteasome activity or interference with PSMB5 expression is beneficial to *C*. *burnetii* intracellular proliferation.

### CirB is required for optimal virulence of *C*. *burnetii* in mice

**Fig 9:**
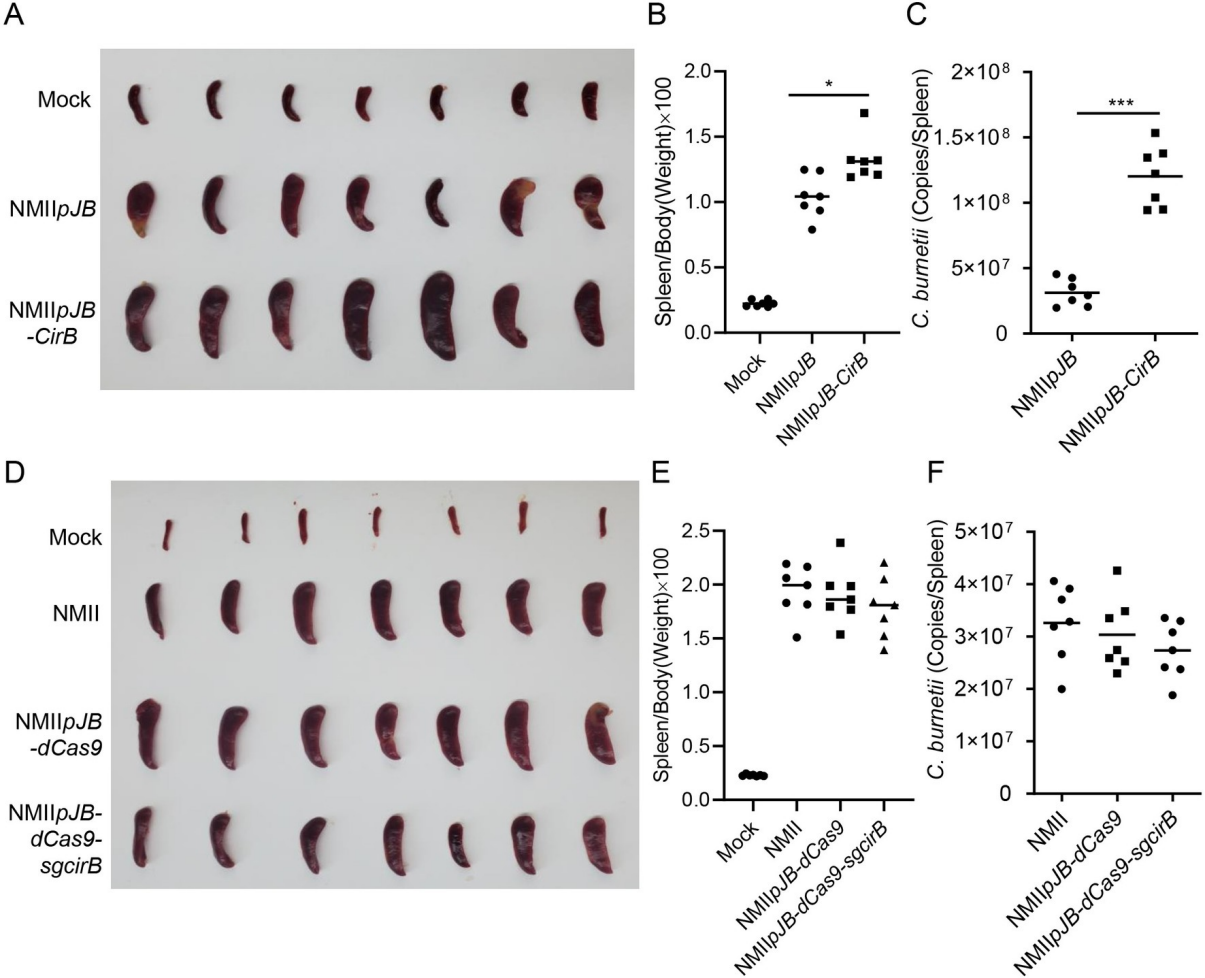
CirB is involved in the pathogenicity of *C*. *burnetii* in a SCID mouse model. (A) SCID mice infected with NMII*pJB* or NMII*pJB-CirB* were sacrificed at 14 days post-infection. The spleens were removed and photographed after bloodletting. (B) The ratio of spleen weight to body weight of each mouse in (A) was calculated. (C) The GE of *C*. *burnetii* in spleens was determined using qPCR. The data are presented as the mean of n = 7 mice per group, and the standard error is indicated by the error bar. (D) Mock-or *C*. *burnetii*-infected SCID mice were sacrificed at 14 days post-infection. The spleens of the mice were removed and photographed after bloodletting. (E) The ratio of spleen weight to body weight of each mouse in (D) was calculated. (F) The GE of *C*. *burnetii* in spleens in (D) was determined using qPCR. The data are presented as the mean of n = 7 mice per group, and the standard error is indicated by the error bar.

To evaluate the role played by CirB in the pathogenicity of *C*. *burnetii*, SCID mice were infected with the aforementioned NMII mutant strains. Two weeks after infection, the mice were sacrificed, and the spleens were removed for weighing and quantification of bacterial load. The spleens of mice infected with NMII*pJB-CirB* were obviously larger than those of mice infected with NMII*pJB* (Fig 9A and 9B). Moreover, the bacterial burden in the spleens of mice infected with NMII*pJB-CirB* was significantly higher than that of mice infected with NMII*pJB* (Fig 9C). However, the degree of splenomegaly in the mice infected with NMII*pdCas9-sgcirB* was not significantly different from that of mice infected with NMII*pdCas9* or NMII (Fig 9D), and no significant difference in splenic bacterial load was detected among the three groups (Fig 9E and 9F). These results indicated that CirB facilitates *C*. *burnetii* proliferation, but it is not an essential virulence factor for SCID mouse infection.

## Discussion

*C*. *burnetii* is an intracellular pathogen of important public health concern as a zoonotic disease and a biological warfare agent [51]. *C*. *burnetii* replicates within enlarged CCVs in host cells and the biogenesis and maintenance of CCVs depend on its Dot/Icm effectors. Despite breakthroughs in genetic manipulation and advances in in vitro culture technology [52,53], little is known about the functions of the majority of *C*. *burnetii* effectors. In line with its ability to form a lysosome-derived vacuole in host cells, autophagy and vesicle transport have been shown to be extensively involved in *C*. *burnetii* infection [11,15,41,54,55]. In addition, the effects of *C*. *burnetii* on host cell apoptosis [7,9] and innate immune signaling pathway [17,18] have recently received considerable attention. With more than 150 effector proteins of *C*. *burnetii* identified, a comprehensive understanding of the host pathways manipulated by these effector proteins is crucial for elucidating *C*. *burnetii* pathogenesis. Our PPI network showed that pathways associated with vesicles and autophagy are, as expected, the main pathways modulated in *C*. *burnetii* infection, which is consistent with previous reports. In addition, information related to host pathways and cellular components, such as the endoplasmic reticulum (ER), noncanonical NF-κB signaling pathway, Akt signaling pathway, and intrinsic apoptosis, is also worthy of further study.

In a previous study by Wallqvist et al [19], Y2H high-throughput screenings were performed to generate *C*. *burnetii*-host PPIs involving 25 *C*. *burnetii* effectors; among these effectors, 12 effectors were also examined using AP-MS in our study (S9A Fig). The 12 interacting pairs initially recognized in the Y2H screen were also verified in our AP-MS assays (S9B and S9C Fig). Coverage between omics level interactomes is often as low as one percent; therefore, the coverage rate we obtained is perfectly acceptable [56]. In addition, some validated interaction pairs reported in previous studies [7,13], such as CvpB-phosphatidylserine (PS) and AnkG-p32, were also validated through our AP-MS analysis, but they had not been identified in the aforementioned Y2H experiments. A phenomenon called assay complementarity was a reasonable explanation for the low coverage between different interaction datasets [56–58]. Generally, Y2H screening detects direct interactions between the bait and prey, while AP-MS method captures weaker interactions and co-complex associations [59]. In addition, protein interactions are complex processes, and the affinity of binding partners or the transient nature of interactions is important, along with the delicate intracellular environment. The reliability of each technique has been a subject of debate [57,58] and the comprehensive analysis of the results strongly influences conclusions drawn. In summary, our AP-MS data provide not only new understanding of *C*. *burnetii*-host interactions but also a comprehensive and orthogonal verification of results reported earlier by others.

Loss of function in CBU0425 has been reported to impair the ability of *C*. *burnetii* to replicate in J774A.1 cells [6]. In our study, knocking down CirB expression using the CRISPRi system exerted no significant effect on the intracellular replication of *C*. *burnetii* in THP-1 cells, and these discrepancies might have resulted from the use of different genetic manipulation methods and cell lines. Nevertheless, we found that the change in CirB expression in *C*. *burnetii* exerted a significant effect on proteasome activity. The establishment of CRISPRi technology in *C*. *burnetii* also will provide an alternative method for in-depth study of effector function and for identifying potential virulence-related effectors of *C*. *burnetii*. A modified version of this CRISPRi system with an inducible promoter is under development.

In addition to the effect of CirB-30aa on the conformational changes of proteasomes detected using AFM (Fig 6F), other possible mechanisms by which CirB affected proteasome activity were also investigated. The level of endogenous PSMB5 was not affected by CirB ectopic expression (S10A and S10B Fig). In addition, we did not detect common posttranslational modifications such as phosphorylation, acetylation or ubiquitination in PMSB5 isolated from cells overexpressing CirB. Furthermore, the mRNA levels of immunoproteasome subunits did not show a significant increase in the presence of *C*. *burnetii* infection-induced inhibition of proteasome activity (S10C and S10D Fig), indicating that *C*. *burnetii* infection did not significantly trigger strong conversion of constitutive proteasomes to immunoproteasomes. Our results suggest that conformational changes of 20S proteasome induced by CirB-30aa likely attribute to the inhibitory effects on proteasome activity. However, we cannot exclude the possibility that the increase in proteasome size is due to the multiple copies of CirB-30aa but not CirB-30aa-mut binding to the proteasome. The exact mechanism by which CirB affects the conformation of the proteasome and the exact amino acid site and structure that bind to the proteasome require further study.

To explore the effect of PSMB5 on the proliferation of *C*. *burnetii* in vivo, *psmb5* gene-knockout mice were generated. Unfortunately, perhaps due to the effect of PSMB5 on sperm motility [60], no *psmb5* gene knockout homozygotes were obtained. Only 4 male *psmb5* gene knockout heterozygotes were available for our experiments. With these mice, we showed that interference with PSMB5 function enhanced *C*. *burnetii* virulence. The underlying mechanisms that link PSMB5 to bacterial virulence may be as the follows. First, as a major component involved in the maintenance of intracellular protein homeostasis, proteasome activity and autophagy complement each other [61,62], and inhibition of proteasome function may increase autophagy flux [63,64], which had long been known to promote the intracellular proliferation of *C*. *burnetii* [54,55]. Second, the proteasome is involved in the degradation of many important signaling molecules. Inhibition of proteasome function by *C*. *burnetii* or CirB blocks IκBα degradation, which may contribute to prolonged suppression of the NF-κB pathway and thus enhanced bacterial intracellular survival. The exact regulatory mechanisms require further investigation.

In summary, our study provided a PPI network between *C*. *burnetii* effectors and human proteins, which will facilitate future investigation of the localization and function of effectors of interest and exploration of the host pathways primarily involved in *C*. *burnetii* infection. Based on the PPIs, we identified CirB as a novel proteasome inhibitor and showed that its inhibitory effect was mediated by a region that spans 91–120 of the protein, which is an effective allosteric inhibitor of the 20S proteasome. This peptide may also be explored as a potential drug for the treatment of diseases associated with abnormal function of the ubiquitin-proteasome pathway.

## Materials and methods

### Ethics statement

All procedures for animal experiments were approved by the Institute of Animal Care and Use Committee (IACUC) of the Academy of Military Medical Sciences (approval no. IACUC -DWZX-2021-026), and all experiments were performed in accordance with the regulation and guidelines of this committee.

### Cell lines, bacterial strains and mice

HEK-293T, HeLa and Vero cells were purchased from ATCC and were grown in DMEM containing 10% fetal bovine serum (FBS). THP-1 cells were purchased from ATCC and cultured in RPMI 1640 medium supplemented with 10% FBS and 0.1% 2- mercaptoethanol. The THP1-CirB, THP1-PSMB5-OE, THP1-PSMB5-KD and THP1-NC cell lines were generated by GenePharma (Suzhu, China) and cultured in the same medium as THP-1 cells with the addition of 1 μg/mL puromycin. All cell lines were cultured at 37°C with 5% CO_2_. The *C*. *burnetii* Nine Mile phase II strain and *C*. *burnetii* Henzerling phase I strain were maintained in our laboratory and cultivated in ACCM-2 with 5% CO_2_ and 2.5% O_2_ at 37°C. NMII*pKM230*, NMII*pTEM1*, NMII*pTEM1-CirB*, NMII*pJB*, NMII*pJB-CirB*, NMII*pdCas9* and NMII*pdCas9-sgcirB* strains were generated by our lab and cultivated in ACCM-2 with the indicated antibiotics. SCID mice (6–8 weeks old) and C57BL/6J mice (6–8 weeks old) were purchased from Vital River Laboratory (Beijing, China). C57BL/6J-*psmb5*^*+/-*^mice were generated by Cyagen Biosciences Inc. (Guangzhou, China) using conventional CRISPR/Cas9 technology. Briefly, the gRNA targeting the exon region of the mouse *psmb5* gene and *cas9* mRNA were coinjected into fertilized mouse eggs to generate targeted knockout offspring. C57BL/6J-*psmb5* ^*+/-*^ mice (heterozygous) were obtained by breeding an F0 targeted mouse with a wild-type C57BL/6J mouse.

### Plasmid construction

To construct eukaryotic expression vectors of *C*. *burnetii* effectors, the genes encoding these effectors were amplified from NMII genomic DNA using primers with vector- homologous sequences. PCR products were cloned into pQM02 plasmid to generate N-terminal-mCherry and C-terminal-twin-Strep fusion proteins. To generate the pJB-dCas9 plasmid, the *dcas*9 gene was amplified from pdCas9-tetO-JtetR plasmid which was kindly provided by Dr. Tong Wang [65], The promoter of *cbu1169* (p1169) was amplified from pJB-Kan-3×FLAG plasmid. The fragment of p1169-*dcas*9 was generated by fusion PCR with the *dcas9* and p1169 segments as templates. The fragment of p1169-RNA scaffold was synthesized by GeneScript (Nanjing, China) and recombined into pJB-Kan-3×FLAG between the *EcoR I* and *Pst I* restriction sites. Similarly, p1169-*dcas*9 fragment was joined to pJB-p1169-sgRNA between the *Pst I* and *Sal I* sites to obtain the final plasmid pJB-dCas9. pdCas9-*sgcirB* was constructed using the same approach, noting that the sgRNA was designed to target *cirB* specifically. The *blaM* gene was amplified and cloned downstream of the 3×FLAG tag in pJB-Kan-3×FLAG to generate the control plasmid pTEM1 used in the β-lactamase translocation experiment. The pTEM1-CirB plasmid was constructed by cloning the *cirB* gene downstream of the *blaM* gene between the *Kpn I* and *Not I* sites. Other plasmids used in this study were constructed by inserting the target sequence into indicated restriction sites of corresponding vectors. All primers used for amplification in this study were shown in S2 Table. The plasmid pKM230 was kindly provided by Dr. Chen Chen [3].

### AP-MS

The pQM02-cbu or pQM02-control plasmids were transfected into 293T cells using Lipofectamine 3000 according to the manufacturer’s instructions, and the expression of the effectors was determined by observing mCherry fluorescence under a fluorescence microscope. Twenty-four hours post transfection, the cells were lysed with lysis buffer (100 mM Tris-HCl, pH 8.0; 150 mM NaCl; 1 mM EDTA; and 1% NP40) supplemented with complete protease and phosphatase inhibitor cocktails, and cell lysates were centrifuged and filtered. The supernatant was collected and incubated with Strep-Tactin Sepharose beads (GE Healthcare, 28-9355-99) at room temperature for 1 hour. Then, the conjugated Strep-Tactin beads were washed with lysis buffer 6 times, and the mCherry-Strep-tagged effectors, as well as the corresponding host binding partners, were eluted with lysis buffer containing 2.5 mM D-desthiobiotin. Each effector protein was expressed and purified in 3 independent experiments. After verification of the successful purification using Western blotting, each purified protein (~100 μg) was freeze-dried, digested and desalted for analysis using LC-MS. The LC-MS/MS analyses were performed with an Orbitrap Fusion Tribrid mass spectrometer (Thermo Fisher Scientific) coupled online to a nanoflow LC system (EASY-nLC 1000 or 1200, Thermo Fisher Scientific). The operation and parameter settings were described previously [66]. The mass spectrometry proteomics data have been deposited to the ProteomeXchange Consortium (http://proteomecentral.proteomexchange.org) via the iProX partner repository [67] with the dataset identifier PXD032380.

### MS data processing

The MS data were searched against the Uniprot Swiss-Prot Human database with the addition of *C*. *burnetii* proteins using Proteome Discoverer software (version 2.4, Thermo Fisher Scientific, Bremen, Germany). The resulting protein group files were first separately correlated. Only the two biological replicates with the closest protein numbers and the highest PCC were retained for the identification of interactions. We downloaded the contaminant repository of all 28 groups of HEK293T cells from the CRAPome [68] and collected the two with the highest spectral count as negative controls to obtain the high-confidence interactions. Finally, 53 effectors and 2 controls were used in the SAINTexpress [69] analysis with the cutoff BFDR< 0.01. Proteins in the top 5^th^ percentile ranked by occurrence rate in CRAPome all human data (443 genes) were removed from the pathogen-host interactions identified by SAINTexpress. The network of high-confidence pathogen-host interactions was visualized using Cytoscape [70].

### Gene Ontology and pathway enrichment analyses

Gene Ontology and pathway enrichment analyses were performed using the R packages clusterProfiler [71] and ReactomePA [72], respectively. The interacting proteins of each effector were analyzed independently. GO terms and pathways with Q-values< 0.05 were considered significantly enriched. Then, the terms and pathways were manually selected for visualization, and dot plots were generated using the R package ggplot2.

### CBU interactor overlap

The overlap between two CBU effectors A and B was measured by the Jaccard-index: J = (A∩B)/(A∪B). J = 0 indicates that no interactions are shared between the two effectors, and J = 1 indicates that all interactions are shared. Then, the Jaccard index incorporated into a matrix is used to draw a heatmap using the R package pheatmap.

### Generation of *C*. *burnetii* mutants

The plasmids described above were electroporated into the *C*. *burnetii* NMII strain with electroporation conditions of 1.8 kV, 25 μF and 500 Ω as previously described [73]. After 3 passages in ACCM-2 with kanamycin (400 μg/mL) or chloramphenicol (4 μg/mL), positive *C*. *burnetii* transformants were screened using ACCM-2 agar plates with the indicated antibiotics. Single colonies of *C*. *burnetii* mutant strains were selected and cultivated for detection. NMII*pKM230* was verified by observing mCherry fluorescence under a microscope. The expression of CirB in NMII*pJB-CirB* or NMII*pTEM1-CirB* strain was verified by Western blotting with an anti-CirB antibody. The expression of *dcas9*, sgRNA-*cirB* and *cirB* genes in the NMII strains carrying CRISPRi plasmids was determined using qRT-PCR, and the expression of dCas9 or CirB in these strains was detected by Western blotting analyses with an anti-Cas9 or anti-CirB antibody, respectively. Before the indicated experiments, the verified positive colonies were screened twice again using the method described above to ensure the singleness and stability of the obtained stains.

### *C*. *burnetii* growth in vitro and in cell lines

All *C*. *burnetii* WT and mutant strains were cultivated in ACCM-2 with or without the corresponding antibiotics. To assess the growth characteristics in ACCM-2, *C*. *burnetii* strains were inoculated into 20 ml of fresh ACCM-2 at the concentration of 1×10^6^ GE/mL, then 10 μl samples of each strain were collected daily for 7 days. The DNA was extracted and subjected to qPCR to determine the GE [74]. To plot the growth curves of *C*. *burnetii* in human macrophages, THP-1 cells were seeded in 12-well plates at the density of 2×10^5^ cells/mL and infected with NMII WT or mutant strains at a multiplicity of infection (MOI) of 20 when the confluence reached 30–50%. THP-1 cells were differentiated into macrophage-like cells after stimulation with 200 nM phorbol myristate acetate (PMA) before infection. Four hours post-infection, cells were washed twice with PBS and continually incubated in fresh medium supplemented with 1% FBS. Then cells were collected at indicated time points for total DNA extraction, and the GE of *C*. *burnetii* was quantitated using qPCR. To detect the proteasome activity or protein expression upon infection, cells were seeded in 96-well plates or 12-well plates at the density of 2×10^5^ cells/mL and infected with *C*. *burnetii* strains at an MOI of 100.

### Immunoprecipitation and Western blotting analysis

To verify the PPIs identified by AP-MS, mCherry-Strep-tagged effectors and FLAG-tagged host proteins were co-expressed in 293T cells. For identification of the interaction between CirB and PSMB5, different tagged CirB and PSMB5 proteins, as well as their truncation mutants, were co-expressed in HeLa cells. Twenty-four hours post transfection, cell lysates were prepared with lysis buffer, and the supernatant of lysates after centrifugation was incubated with 2 μg of anti-FLAG antibody (Sigma, F1804) at 4°C for 6 hours. Then, 50 μl of a 50% slurry consisting of Protein A/G plus-agarose (GE Healthcare, 17061801) was added, mixed and incubated for another 6 hours or overnight. After 5 washes with lysis buffer, the beads were boiled with 2×loading buffer for 10 min. The samples were fractionated by electrophoresis on SDS-PAGE gels, and the resolved proteins were transferred to polyvinylidene difluoride membranes. After blocking with 5% skim milk, the membranes were incubated with the indicated antibodies, followed by appropriate secondary antibodies. Blots were developed with an enhanced chemiluminescence (ECL) kit.

### Confocal laser scanning

To observe the subcellular distribution of the *C*. *burnetii* effectors, HeLa cells were seeded on coverslips in 24- well plates and cultured overnight and then transfected with pQM02-cbu plasmids. Twenty-four hours post transfection, the cells were fixed with 4% paraformaldehyde and blocked with 1% bovine serum albumin. Then, LAMP1 was stained with anti-LAMP1 antibody (CST, 9091S), followed by goat anti-rabbit IgG Alexa Fluor 488 antibody (Abcam, ab150113), and the nucleus was stained with DAPI. To detect the colocalization of exogenous CirB and PSMB5 truncation mutants, HeLa cells were transfected with plasmids expressing CirB or its truncation mutants and plasmids expressing PSMB5 or its truncation mutants. After fixation and permeabilization, cells were blocked and probed with an anti-FLAG or anti-HA antibody (Abcam, ab9110), followed by treatment with a goat anti-mouse IgG Alexa Fluor 488 antibody or goat anti-rabbit IgG Alexa Fluor 594 antibody (Abcam, ab150116). After 3 washes with PBS, the cell nuclei were stained with DAPI. To determine the relative localization of CirB and PSMB5 during infection, HeLa cells were seeded on coverslips on 24-well plates and transfected with pQM02-CirB. Four hours later, the cells were infected with *C*. *burnetii* at an MOI of 100. Two days post-infection, cells were fixed and probed with an anti-PSMB5 antibody (Thermo, PA1-977) and anti-*C*. *burnetii* serum, followed by appropriate secondary antibodies and DAPI. The coverslips were removed and mounted on slides with Prolong Gold antifade mounting solution (Thermo, p36935). Samples were observed with a spinning disk confocal microscope (Nikon Ti-Eclipse microscope).

### Detection of intracellular proteasome activity

Cells were cultured in a 96-well plate at the density of 1×10^4^ cells/well and subjected to plasmid transfection or *C*. *burnetii* infection. At the appointed time points, the cells were detected using a proteasome activity detection kit (Promega, G8660). Briefly, the assay buffer was prepared by dissolving luciferin detection reagent into the Proteasome-Glo buffer. Then, the Suc-LLVY-Glo substrate was added at the ratio of 1:200 to generate the reaction reagent. After 30 min of incubation at room temperature, 100 μl of the reaction reagent were added to the cells, and the luminescence of each sample was measured in a plate reading luminometer after an incubation for a minimum of 10 minutes.

### Detection of proteasome activity in vitro

A final volume of 100 μl of 50 mM Tris-HCl (pH 8.0) was added to a 96 well-plate with 0.01% SDS, purified 20S proteasome (Enzo, BML-PW8720) at a final concentration of 0.001 mg/mL and peptide stock solutions at different concentrations (0.05, 0.1, 0.2, 0.5, 1 and 10 mM). Suc-LLVY-AMC (Enzo, BML-P802) substrate was added at 20 μM final concentration, and the fluorescence was measured continuously every 5 minutes for 60 minutes.

### TEM translocation assays

TEM translocation assays were performed as described previously [75] with some modifications. Briefly, differentiated THP-1 cells were used for the *C*. *burnetii* TEM translocation assay. Cells were propagated in RPMI 1640 medium containing 10% FBS and 0.1% 2-mercaptoethanol and seeded in a 96-well plate at 5×10^4^ cells/well 48 h before infection. *C*. *burnetii* strains carrying β-lactamase fusions were applied at an MOI of 100 to infect THP-1 cells. Four hours after infection, cells were washed with PBS to remove uninternalized bacteria, and 20 μl of 6×CCF4/AM fluorescent substrate in solution was added at 24 hours post-infection and continuously incubated at room temperature for 1–3 hours before fluorescence detection. Samples were observed under a fluorescence microscope (Olympus), and positive cells were marked blue.

### AFM

The AFM images were collected in tapping mode (Nanoscope III, Digital Instruments) as described previously [46,76]. Briefly, 1 μl of human 20S proteasome with or without 10 μM CirB-30aa or CirB-30aa-m in 4 μl of 5 mM Tris-HCl (pH 7.0) was deposited on a freshly cleaved mica surface. After 1 hour of incubation and attachment at room temperature, the samples were overlaid with 100 μl of 5 mM Tris-HCl buffer in a wet chamber, and imaged using oxide-sharpened silicon nitride tips with a spring constant of 0.1 N/m. Fields ranging from 0.09 to 1 μm^2^ containing multiple proteasome particles were continuously scanned with JPK Nonwizard 4 software. The parameters set for scan were as follows: 0.1 Nm for the setpoint, 100 nm for the Z length, 20 μm/s for the Z speed and 12.5 ms for the pixel time. The acquired images were analyzed with JPKSPM Data Processing software.

### Quantitative analysis of *C*. *burnetii*

Total DNA was extracted from *C*. *burnetii* or *C*. *burnetii*-infected cells using a DNeasy Tissue kit following the manufacturer’s instructions. Real-time PCR reactions were performed in duplicates with Taqman Universal Master mix on an Applied Biosystems QuantStudio 3. The GE of *C*. *burnetii* was quantified using the diluted pQGK-Com1 plasmid as the standard sample. Primers and probes used for quantification were as follows: *com*1-F: AAAACCTCCGCGTTGTCTTCA; *com*1-R: GCTAATGATACTTTGGCAGCGTATTG; *com*1-probe: AGAACTGCCCATTTTTG GCGGCCA.

### Animal experiments

In a SCID mouse infection model, 6-week-old female SCID mice (n = 6) were treated with MG132 (10 μg/kg) daily [77,78] or PBS via intraperitoneal (i.p.) injection. Two weeks later, the mice were infected with 5×10^6^ viable *C*. *burnetii* phase II strain via i.p. injection. Fourteen days post-infection, the mice were sacrificed, and spleens were removed and weighed to determine the degree of splenomegaly (spleen weight/body weight). The *C*. *burnetii* GE in organs was also determined using qPCR. For *psmb5*^*+/-*^ mice (n = 4), 1×10^6^ viable *C*. *burnetii* phase I strain was administered via i.p. injection in an animal biosafety level-3 laboratory (ABSL-3). Fourteen days post-infection, mice were sacrificed and relevant data were collected as described above. To examine the effect of CirB on *C*. *burnetii* pathogenicity, SCID mice (n = 7) were infected with NMII, NMII*pJB*, NMII*pJB-cirB*, NMII*pdCas9* or NMII*pdCas9-sgcirB* at the dose of 1×10^7^ GE/mouse. After 14 days, mice were sacrificed and the bacterial load was quantified.

### Statistical analysis

Statistical significance of differences in the *C*. *burnetii* GE, band density, ratio of spleen or liver weight to body weight, fluorescence intensity and width of the proteasome was determined using Student’s t test, one-way or two-way variance (ANOVA). The data were analyzed using GraphPad Prism 8.0 software (GraphPad). For all analyses, a *p* value < 0.05 was deemed to be significant.

## Supporting information

S1 Fig Localization of some effectors and the Pearson’s correlation coefficients of biological replicates. (TIF)

S2 Fig Coimmunoprecipitation verification of the PPIs screened by AP-MS. (TIF)

S3 Fig Interaction pairs clustered in different pathways. (TIF)

S4 Fig MTT assays and examination of protein expression and proteasome activity of purified proteasomes. (TIF)

S5 Fig Detection of related characteristics of *C*. *burnetii* mutants. (TIF)

S6 Fig Identification of the interacting domains of CirB and PSMB5. (TIF)

S7 Fig The effect of PSMB5 expression on *C*. *burnetii* intracellular replication. (TIF)

S8 Fig The effect of MG132 stimulation on mice and the detection of *psmb5*^*+/-*^ mice. (TIF)

S9 Fig Overlaps between Y2H and AP-MS results. (TIF)

S10 Fig The effect of CirB on PSMB5 expression. (TIF)

S1 Table Analyses of AP-MS data. (XLSX)

S2 Table Primers used for amplification. (XLSX)
