# Supplementary figures and images for: A protein–protein interaction map reveals that the Coxiella burnetii effector CirB inhibits host proteasome activity
Source: PLoS Pathog. 2022 Jul 11;18(7):e1010660. doi: 10.1371/journal.ppat.1010660 (PMC9273094; doi:10.1371/journal.ppat.1010660)

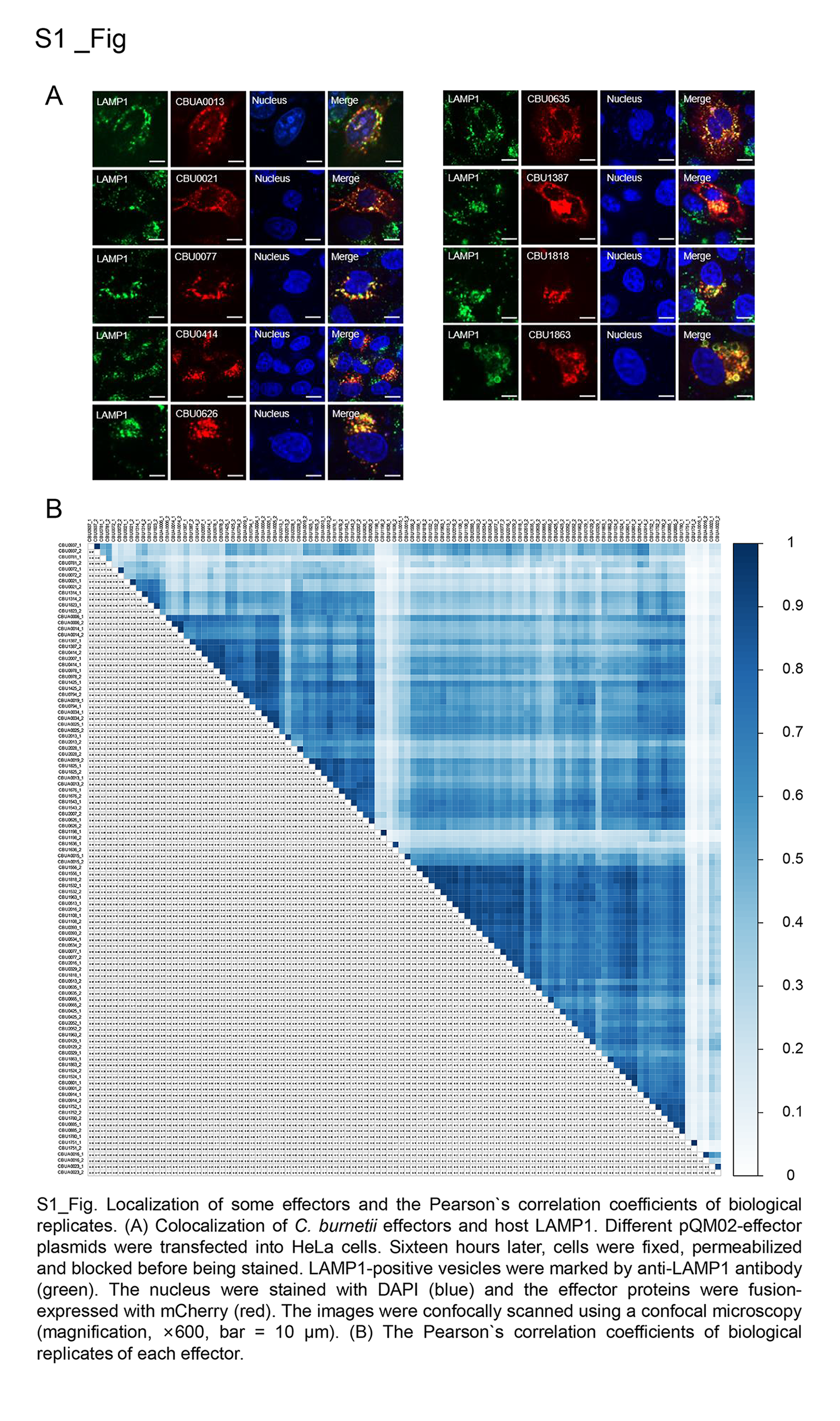

Supplement: S1 Fig — (TIF) [file ppat.1010660.s001.tif]

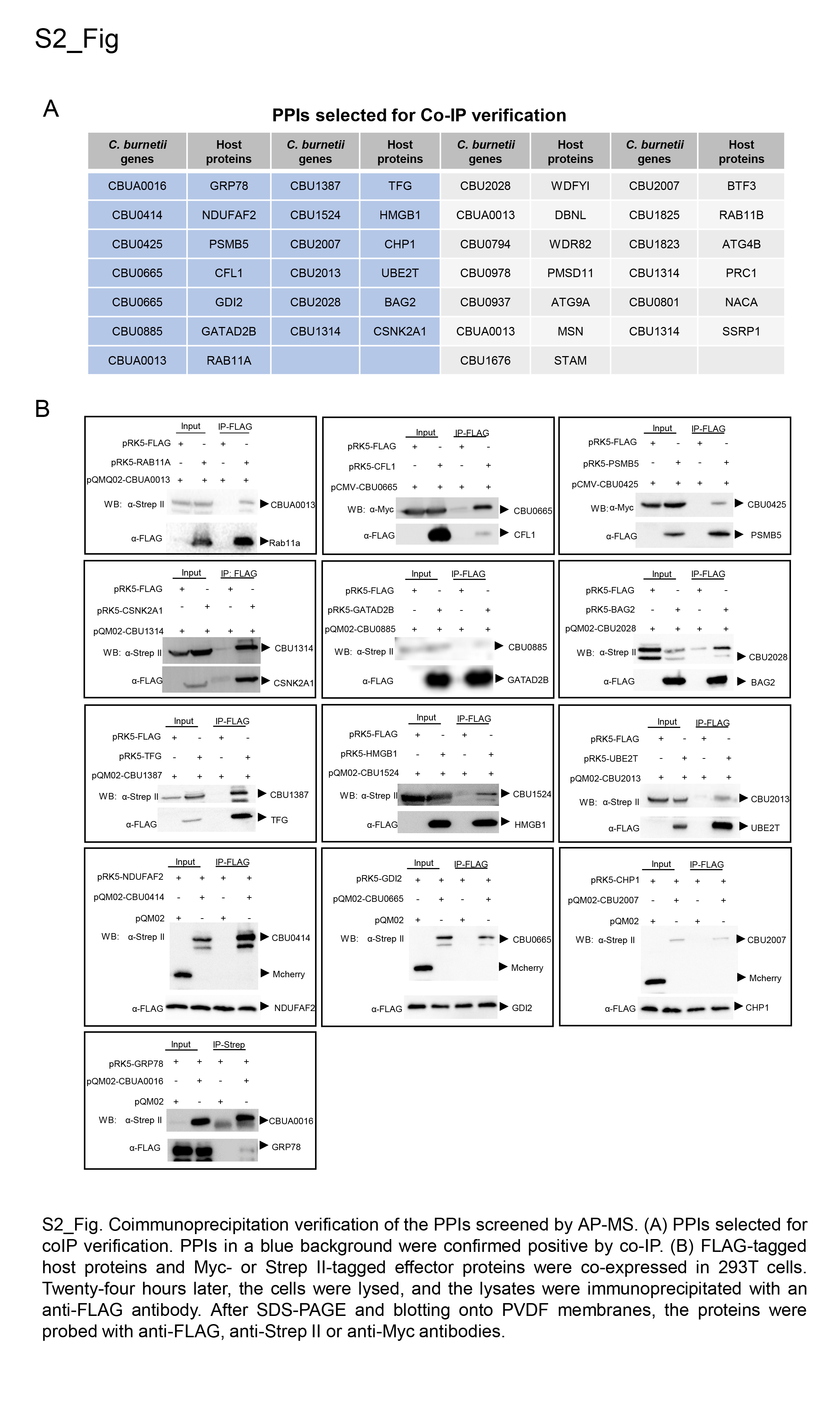

Supplement: S2 Fig — (TIF) [file ppat.1010660.s002.tif]

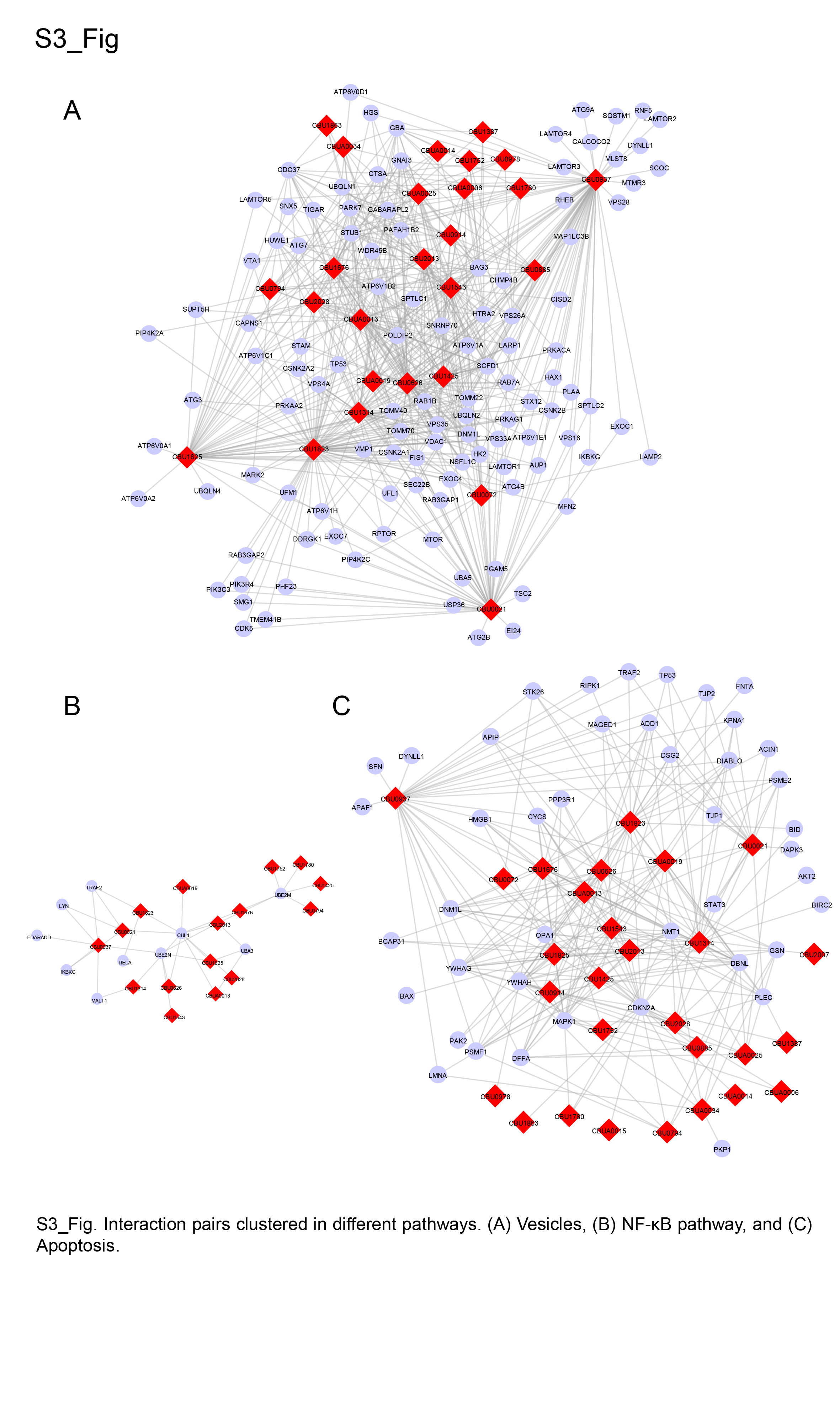

Supplement: S3 Fig — (TIF) [file ppat.1010660.s003.tif]

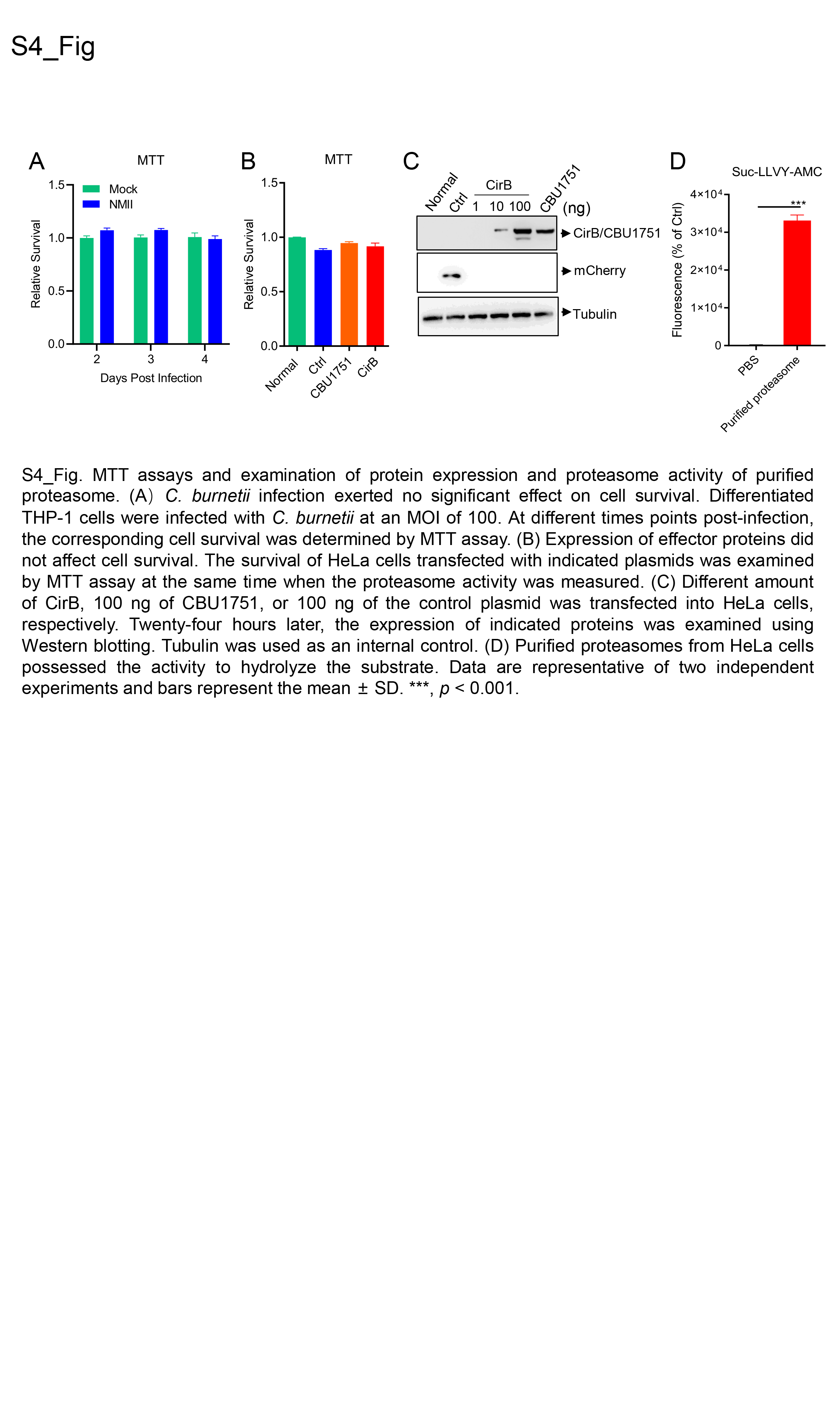

Supplement: S4 Fig — (TIF) [file ppat.1010660.s004.tif]

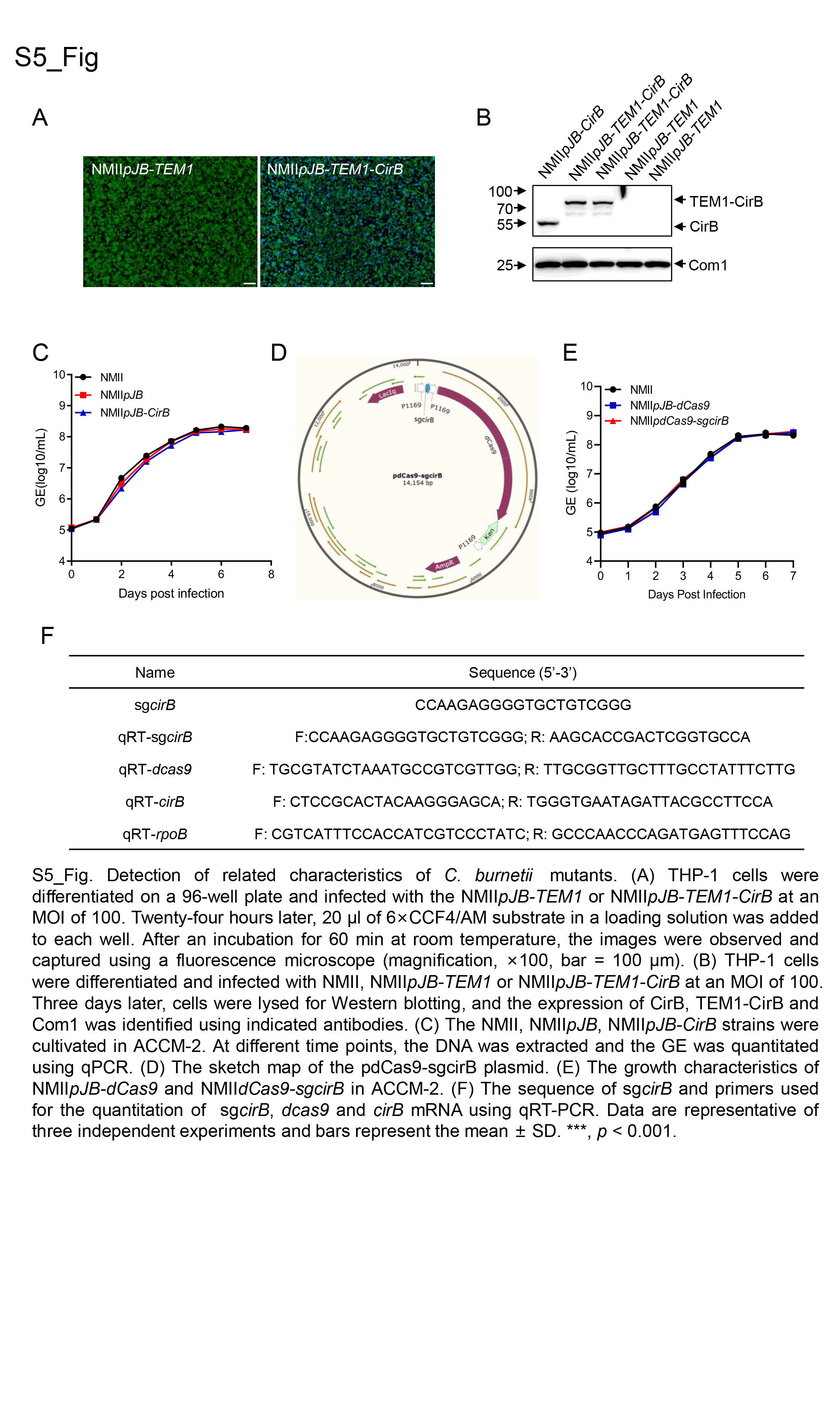

Supplement: S5 Fig — (TIF) [file ppat.1010660.s005.tif]

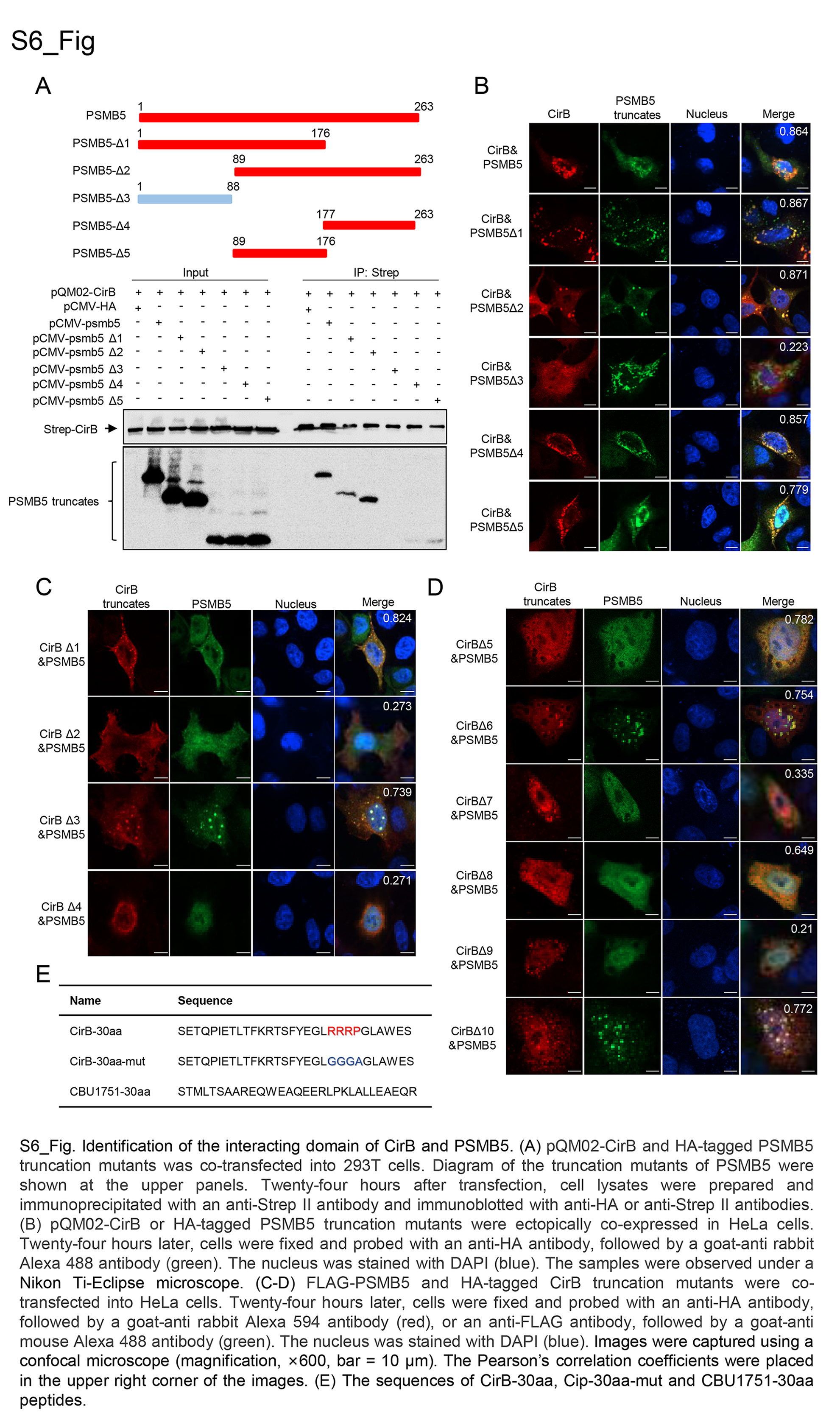

Supplement: S6 Fig — (TIF) [file ppat.1010660.s006.tif]

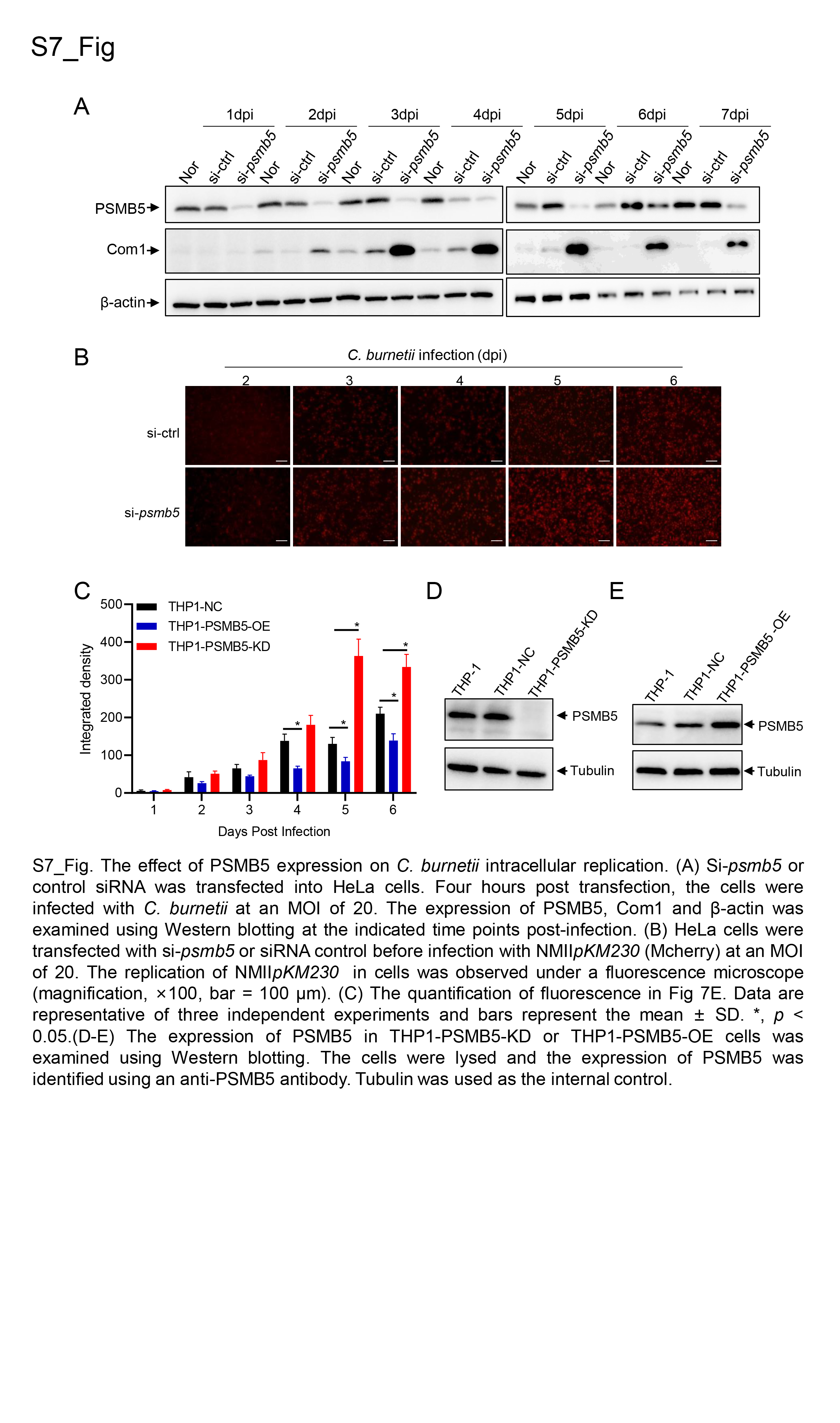

Supplement: S7 Fig — (TIF) [file ppat.1010660.s007.tif]

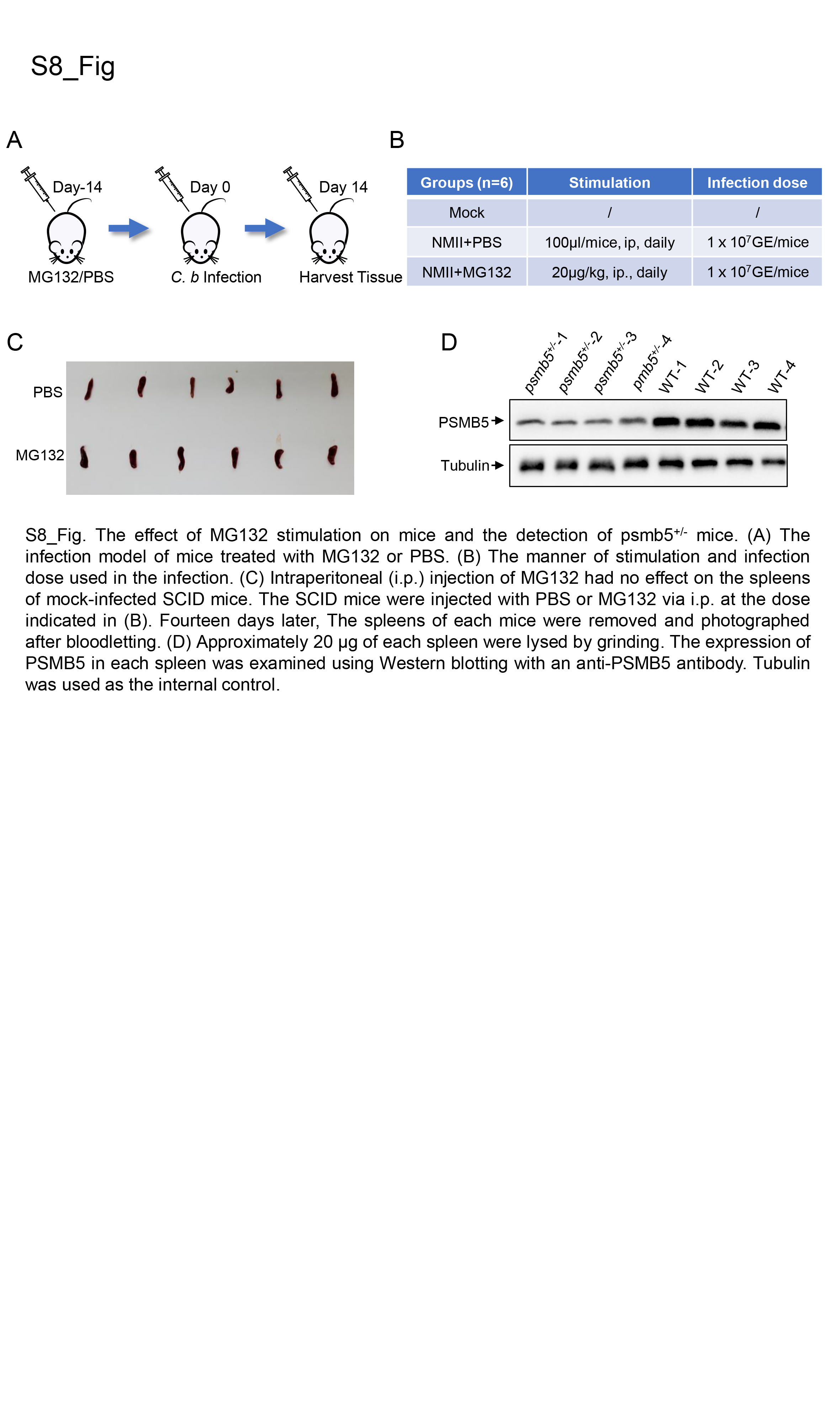

Supplement: S8 Fig — (TIF) [file ppat.1010660.s008.tif]

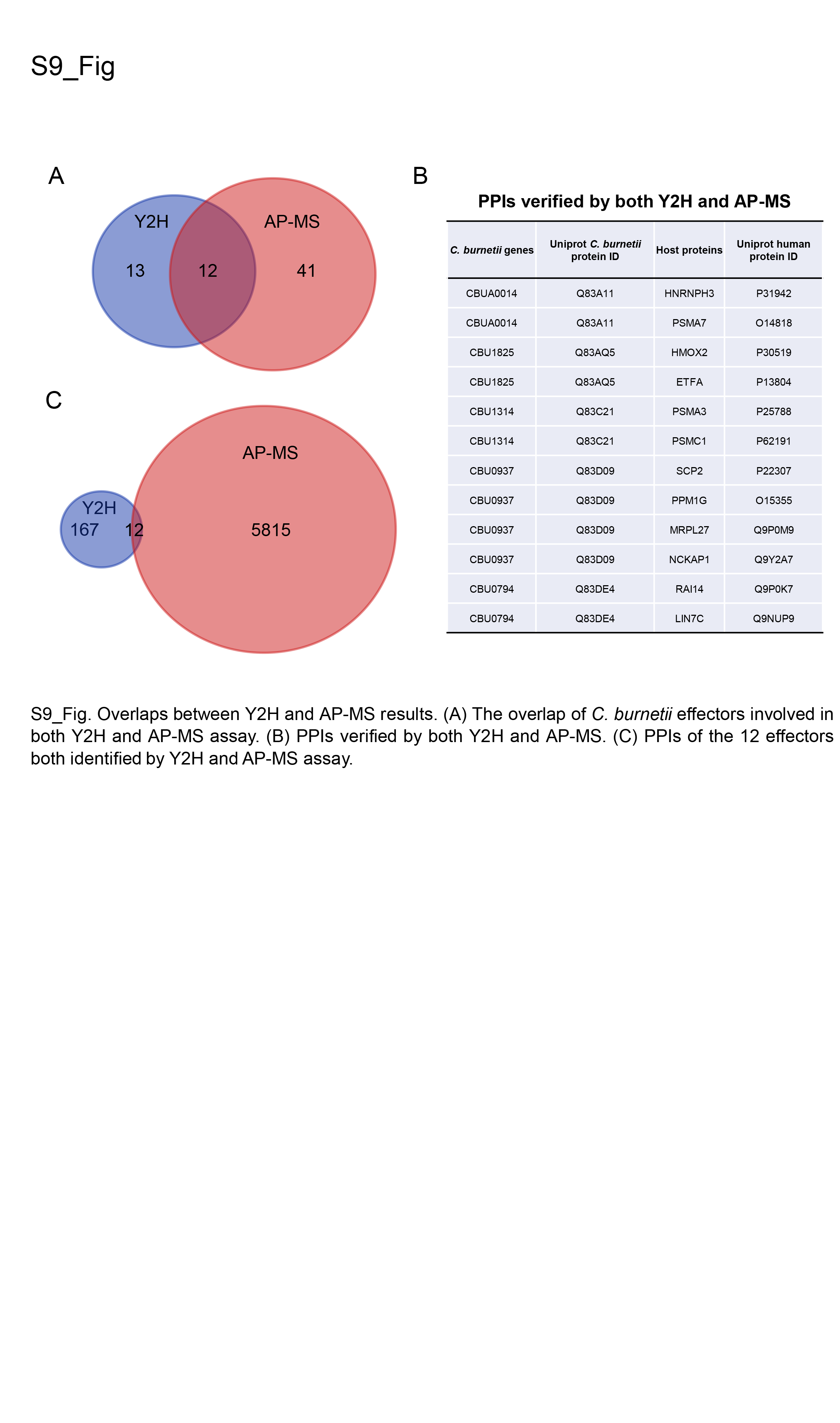

Supplement: S9 Fig — (TIF) [file ppat.1010660.s009.tif]

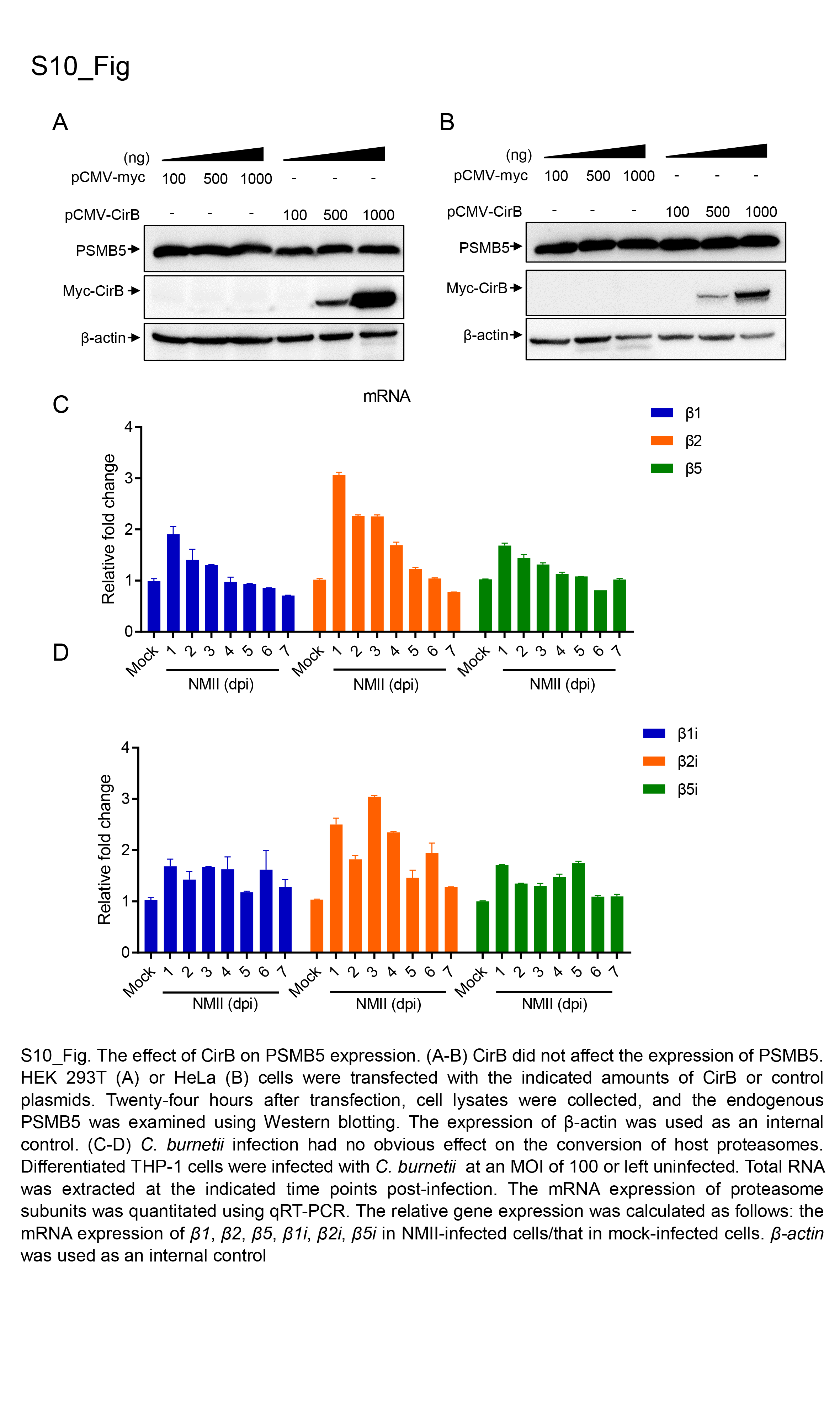

Supplement: S10 Fig — (TIF) [file ppat.1010660.s010.tif]
